# Direct electronetting of high-performance membranes based on self-assembled 2D nanoarchitectured networks

**DOI:** 10.1038/s41467-019-09444-y

**Published:** 2019-03-29

**Authors:** Shichao Zhang, Hui Liu, Ning Tang, Jianlong Ge, Jianyong Yu, Bin Ding

**Affiliations:** 1 State Key Laboratory for Modification of Chemical Fibers and Polymer Materials, College of Textiles, Donghua University, Shanghai, 201620 China; 2 Innovation Center for Textile Science and Technology, Donghua University, Shanghai, 200051 China

**Keywords:** Synthesis and processing, Two-dimensional materials, Polymers

## Abstract

There is an increasing demand worldwide on advanced two-dimensional (2D) nanofibrous networks with applications ranging from environmental protection and electrical devices to bioengineering. Design of such nanoarchitectured materials has been considered a long-standing challenge. Herein, we report a direct electronetting technology for the fabrication of self-assembled 2D nanoarchitectured networks (nano-nets) from various materials. Tailoring of the precursor solution and of the microelectric field allows charged droplets, which are ejected from a Taylor cone, to levitate, deform and phase separate before they self-assemble a 2D nanofibre network architecture. The fabricated nano-nets show mechanical robustness and benefit from nanostructural properties such as enhanced surface wettability, high transparency, separation and improved air filtration properties. Calcination of the nano-nets results in the formation of carbon nano-nets with electric conductivity and titanium dioxide nano-nets with bioprotective properties.

## Abstract (web summary)

Nanofibrous networks are applied in environmental protection and electrical or bioengineering and experience a great demand, but the design of these functional materials is challenging. Here the authors demonstrate an electronetting technology for fabricating self-assembled nanonets from various materials.

## Introduction

One-dimensional (1D) nanomaterials are attractive for their wide range of applications in the fields of environment, electronics, optics, energy and healthcare^1–3^. The high-performance regime currently contains very few forms: nanotubes, nanowires, nanorods and nanofibers^4–7^. All these ultrafine materials have characteristic sizes below 100 nm, which bring about the structural advantages of high surface-to-volume ratios and unique electronic, optical, thermal and mechanical properties in comparison to conventional materials. The story is, however, not entirely nanoscale, but eventually includes macroscopic bulk structures in the form of two-dimensional (2D) membranes or three-dimensional (3D) bulks assembled together as basic functioning units^8–10^. Nature offers a simple yet efficient idea associated with material assembly: creating a continuous fibrous network can substantially improve material utilization and the resultant properties^11,12^. This effect is vividly demonstrated in cases such as dragonfly wings, spider webs and honeycombs, which possess a network architecture of ultrafine fibers but are structurally robust and multifunctional^8,13^. Inspired by such biostructures, assembling 2D nanofibrous networks with high continuity and porous structures from 1D nanomaterials could be an effective strategy for maximizing their performance to facilitate widespread applications. Many assembly technologies, including deposition, self-assembly, in situ growth, etc. have been developed and used to assemble nanotubes, nanowires or nanorods into bulk materials^14–17^. However, the inherent limits on the continuity (usual length: nanotubes <100 μm, nanowires <200 μm, and nanorods <10 μm) and diversity of these nanoscale building blocks, combined with the lack of structural integration and precise control of the assembly architecture, present major challenges in reducing the performance degradation from nanostructures to their assembled bulk materials.

Electrospinning technology enables the easily scalable synthesis of continuous nanofibers from various materials (polymer, metal, ceramic, carbon, etc.), and the resultant nanofibers combine remarkable continuity (up to kilometers), robust mechanical properties and fine flexibility^18–21^. Despite these superior features, the pervasive problem associated with electrospun nanofibers is their pseudo-nanoscale diameters of >100 nm (usually 0.2–2 μm) and random spinning-deposition manner. The resulting nanofibers show limited performance improvement in terms of surface area, pore structure and porosity due to pseudo-nanoscale structures, and commonly assemble into lamellar-deposited nonwovens rather than into controlled and organized assemblies^22,23^. Moreover, they usually lack stable cross-linking between stacking nanofibers, and thus exhibit poor mechanical strength. Our previous work prepared 2D nanowebs with nanoscale diameters (20–50 nm) and topological Steiner-tree structures from several kinds of special polymeric materials using electrospinning/netting technique^24,25^. In addition to addressing the fundamental challenge in electrospun nanofibers, like fiber diameter and connectivity, the 2D nanowebs overcome limitations of most existing fibrous networks (for example, networks from carbon or cellulose nanocrystal films coated grids after polymer/solvent evaporation), including limited porosity and uncontrolled pore size due to non-uniform and partly porous substrate-like structure, and low mechanical property and insufficient size required for their effective applications^26,27^. The resultant nanoweb materials, in this regard, hold great promise as exceptional candidates for applications in sensors, air filtration and protective clothing^28–30^. Despite the obtained performance improvement, these nanowebs are only an occasionally formed accessory product accompanying the formation of electrospun nanofibers, which occur in a discontinuous and small piece form (micrometer-scale pieces, random distribution in the membrane and a low coverage rate <10%), with the mass ratio of nanowebs in the whole nanofiber/web membrane being less than 2%^24,25,28^. The challenge, therefore, is to develop an innovative technology to create integrated, continuous and ordered nanofibrous networks capable of forming a mechanically robust 2D porous assembly, without compromising the unique properties of the intrinsic fibers with true nanoscale diameters (<100 nm).

Herein, we demonstrate a methodology for creating 2D self-assembled networks that consist of nanoscale fibers (10–40 nm), which we call nano-nets. The premise of our design is that the ejection, levitation, deformation and phase separation of the droplets from a Taylor cone are effectively controlled, using unique direct electronetting, to assemble 2D network architectures with desirable nanostructural features and tunable bulk properties on a large scale. We show that the nano-net membranes exhibit the integrated properties of highly porous structures with small pore sizes while maintaining ultrathin thickness, robust mechanical strength, enhanced surface wettability, high transparency and superior functionality for applications of air filtration, liquid separation, electric conduction and bioprotective activities, which all benefit from the synergistic effect of nanoscale fibers and well-controlled network assembly.

## Results

### Assembly model of 2D nano-nets

**Fig. 1:**
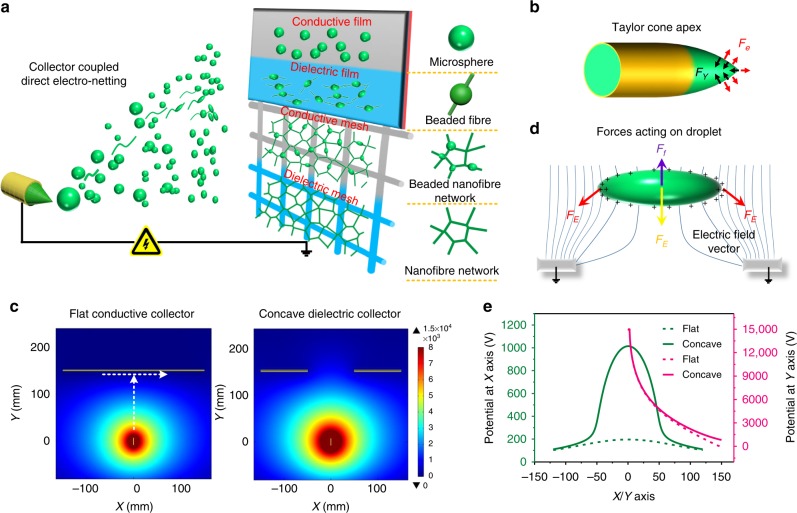
Origin, evolution and regulation of two-dimensional (2D) nano-nets during direct electronetting. **a** Schematic showing the collector inductively coupled direct electronetting strategy. Four designed collectors trigger the assembly of four different architectures. **b** Competition between the two main forces (Coulomb repulsion *F*_e_ and hydrostatic pressure *F*_γ_) on the Taylor cone apex. **c** Electric field optimization by customizing the collectors in terms of conductivity and configuration. Left, flat conductive collector. Right, concave dielectric collector. **d** Forces acting on the charged droplet flying across a concave collector. Electrostatic force *F*_E_ and air drag *F*_f_. **e** Profiles of the potential vs distance of electric fields generated using the two collectors in (**c**). The *X-* and *Y*-axis directions are shown using the horizontal and vertical arrows at the left of (**c**), respectively

We proposed a collector inductively coupled direct electronetting technique to assemble various micro-/nanoarchitectures including microspheres, beaded fibers, beaded nanofiber nets and nano-nets (as illustrated in Fig. 1a). The electrostatic field can be precisely controlled at the micro level to achieve on-demand distribution by tailoring the dielectric properties (conductive or dielectric) and configurations (flat or concave) of the collectors. The unique direct electronetting process was performed using extremely diluted (1–3 wt%) precursor solutions of high-molecular-weight polymers. The polymer concentrations of these solutions were far below that for electrospinning, and their molecular weights were too high to meet the requirement for electrospraying^31–33^. This is the principal feature in terms of raw materials that differentiates direct electronetting from other electrohydrodynamic techniques. To reveal the self-assembly of the obtained architectures, we developed an ejection model for the Taylor cone, which was capable of linking the solution properties and operating parameters to the forms and dynamics of the issued fluids. Two distinct ejection modes (i.e., jet and droplet) driven by the competition between Coulombic repulsion *F*_e_ and hydrostatic pressure *F*_γ_ at the Taylor cone apex were proposed, and the schematic model is shown in Fig. 1b. More excitingly, we can easily predict these two ejection modes using our two critical equations obtained based on force preconditions and theoretical derivation (Supplementary Note 1). The increase in the charge density of the liquid caused the ejection mode of the Taylor cone to change accordingly: cylindrical jets formed when the charge density exceeded the jet threshold of *J*_c_, while spherical droplets were ejected with a higher charge density than the droplet threshold of *D*_c_^24,25^.

1 $$J_{\mathrm{{c}}} = \sqrt {{\mathrm{64\varepsilon \gamma }}/\delta \rho ^2D^3}.$$

2 $$D_{\mathrm{{c}}} = \sqrt {{\mathrm{288\varepsilon \gamma /}}\delta \rho ^2D^3}.$$

Collectors with different permittivities usually allow the electrostatic field to become an area where the potential exhibits changed distribution states and, thus, greatly affects the dynamics of the Taylor cone. Herein, we introduce a correction factor (*δ*) to include the impact from receivers into our ejection model to reveal the relationship between substrate properties and electric potential during direct electronetting (Supplementary Note 1).

The dynamic evolution of the microsized droplets was driven by their surrounding electric field^25,34–36^. Here, we performed finite element method simulations using COMSOL^®^ Multiphysics software with an AC/DC module to simulate the electrostatic field (Supplementary Table 2 and Supplementary Methods), analyze its capacity for tailoring the ejected liquid configuration (jet or droplet) and, thus, guide the actual operation for direct electronetting. Figure 1c and Supplementary Fig. 1 illustrate the distribution of electric fields using different collectors with designed conductivities and configurations. In dramatic contrast to that of the flat conductive collector, the electric field becomes significantly concentrated due to polarization of the dielectric collector, resulting in a slower damping speed of the electric potential along the *Y*-axis. Meanwhile, the concave configuration leads to a significant fluctuation of electric potential along the *X*-axis, and this fluctuation functions as an insulating-block parallel receiver to allow the forces to drive the deformation of the charged droplets. The schematic showing the forces acting on the droplets in such a microelectric field is illustrated in Fig. 1d. Similar to the patterning of electrospun nanofibers, the electrostatic force along the potential gradient combined with the synergistic effect from air drag allows the stretching of charged droplets and evolution of their architecture from microspheres to fibers after solvent evaporation^37,38^. To further quantitatively analyze the electric field, COMSOL^®^ was used, and the resultant potential-distance curves of two contrastive systems are shown in Fig. 1e. Obviously, the topographically structured collectors with different conductivities enabled precise control of the electric field distribution. Using a concave dielectric receiver, the potential across the collector (*X*-axis) showed greatly enhanced fluctuation of 500% (from ~200 to ~1000 V) compared to the almost-unchanged potential of ~200 V using a flat conductive collector, and this result further confirmed the capacity of the inductively coupled collectors for controlling the evolution of charged droplets. Moreover, in contrast to the flat conductive collector, a sustained higher potential along the *Y*-axis was achieved using the dielectric collector, and thus it can greatly facilitate droplet ejection from the Taylor cone.

Mother Nature’s legacy not only makes Voronoi cells visible in nature, but shows ordered dynamic evolution phenomena as well^39–42^. Our obtained nano-nets may be regarded as another similar case, and their unique 2D nanoarchitectured fibrous networks has never before been reported (Supplementary Fig. 2). Here, we proposed a possible formation process for 2D nanofibrous networks (Supplementary Fig. 3 and Supplementary Discussion). Using the direct electronetting, the charged droplets, which could be regarded as a levitating cluster, perhaps undergo rapid self-assembly of their spatial position based on dissipative effect to achieve an energy/material minimum state^40,42,43^, and rapid stretching deformation due to the differential microelectric fields^37,38,44^, as clarified in Fig. 1d. Thus, further stretching and solvent evaporation would result in the formation of nanofiber assemblies with ideal or weighted 2D networks (i.e., nano-nets or beaded nanofiber nets). Evidence of this possible formation process also arose from the following scanning electron microscopy (SEM) observation of the architectures obtained using different topographically structured collectors with designed conductivities.

### Model validation, design and processing of 2D nano-nets

**Fig. 2:**
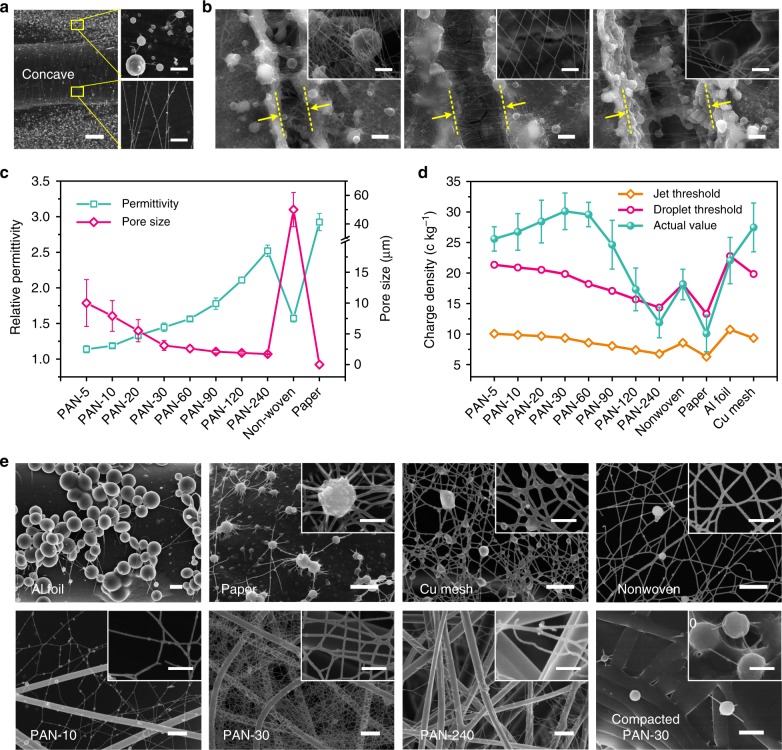
Model validation, design and synthesis of the self-assembled architectures. **a** Scanning electron microscopy (SEM) images of polyvinylidene fluoride (PVDF) self-assembled architectures on the flat collector with a concave zone. Scale bar at the left of (**a**), 10 μm. Scale bars at the right of (**a**), 1 μm. **b** SEM images of PVDF self-assembled architectures across concaves with different widths. The concave widths in (**b**) (from left to right) are 3, 5 and 10 μm. Scale bars in (**b**), 2 μm. Scale bars in the insets of (**b**), 500 nm. **c** Relative permittivity of the collectors including polyacrylonitrile (PAN) fiber membranes with different electrospinning durations, nonwoven fabric and paper. PAN-X, X is the spinning duration (min). **d** Charge density (*e*/*m*), jet and droplet thresholds of the fluids ejected from the PVDF solution and received using various collectors in (**c**) during the direct electronetting process. Error bars in (**c**, **d**) represent s.d. **e** Typical SEM images of PVDF self-assembled architectures collected using different collectors. Top images in (**e**) (from left to right): aluminum (Al) foil, nonporous paper, copper (Cu) mesh, nonwoven fabric. Bottom images in (**e**) PAN fiber membranes with various structures (from left to right): untreated 10 min, untreated 30 min, untreated 240 min and compacted 30 min. The insets are the corresponding high-magnification images of the self-assembled architectures. Scale bars in (**e**), 1 μm. Scale bars in the insets of (**e**), 500 nm

To elaborate the structural evolution and control of the self-assembled architectures obtained using direct electronetting, we first chose polyvinylidene fluoride (PVDF) system as a model for our proof-of-concept study, in considering its outstanding properties such as high thermal/chemical stability, robust mechanical property and strong piezo-, pyro-, ferroelectric properties for use in various applications^7,45^. The detailed parameters for PVDF sample preparation for SEM observation are illustrated in Supplementary Table 2. First, a concave aluminum (Al) foil was used to collect the self-assembled architectures, and the observed SEM result is shown in Fig. 2a. Obviously, different surface topographies led to the formation of different architectures. Only microspheres formed on the flat zone (top right image in Fig. 2a), and this phenomenon agreed very well with the prediction of our proposed model. In contrast, 1D nanofibers were deposited across the concave on the collector (bottom right image in Fig. 2a), which provided strong evidence to confirm the stretching deformation of droplets due to the potential gradient in the microelectric field (Fig. 1c–e). To further reveal the function of the surface topography of collectors, we create concaves with different widths on dielectric polymethyl methacrylate films using femtosecond laser ablation; the fibrous materials obtained using such collectors are shown in Fig. 2b. Beaded fibers assembled in the flat zone on both sides of the concaves, while nanofibers and nano-nets formed across the concaves. Further analysis of the architectures on the concave revealed that upon increasing the concave width from 3 to 5 μm, the formation of beaded fibers was greatly inhibited, while the self-assembly of nano-nets was significantly enhanced, as shown in Fig. 2b (insets). However, further increasing the concave width to 10 μm caused the nano-nets to be broken due to the excessive stretching of charged droplets (inset of Fig. 2b). This change suggests an obvious size match between droplet deformation and the microelectric field, providing another strategy to tailor the self-assembly of 2D nano-nets.

To adjust the microelectric field during direct electronetting process, various topographically structured collectors (flat or porous) with designed conductivities (conductive or dielectric) were used. In addition to the common substrates, such as nonwoven fabric, paper, Al foil and Cu mesh, we also employed a polyacrylonitrile (PAN) electrospun fiber membrane as a flexibly adjustable collector. Figure 2c presents the relative permittivities and pore sizes of the used substrates. Upon increasing the spinning duration from 5 to 240 min, the permittivity of PAN fiber membranes gradually increased from 1.13 to 2.52, while their pore size decreased significantly from ~10 to ~1.7 μm. This change in the PAN membranes could effectively fill the gap between nonwoven fabric (permittivity of 1.57 and pore size of ~50 μm) and nonporous paper (permittivity of 2.92) to achieve a continuous design for inductively coupled collectors. Moreover, by plugging the solution properties and process parameters into our assembly model (Supplementary Table 3), we established a numerical prediction diagram for the formation of 2D nano-nets. The critical formation thresholds for jets and droplets using various substrates were calculated, and their actual charge densities were experimentally measured^25^. The obtained results are shown in Fig. 2d. Obviously, the increased dielectric property allows for significantly decreased jet and droplet thresholds. This result was ascribed to the concentration of the electric field due to the dielectric collectors (Fig. 1c–e), which could generate an induced charge in the electrostatic field. In contrast, using conductive Al foil and Cu mesh caused much higher jet and droplet thresholds. For instance, the droplet thresholds of the fluids using Al foil and Cu mesh collectors were 22.7 and 19.8 c kg^−1^, while those using the dielectric collectors were much lower: 18.1 c kg^−1^ for nonwoven fabric, 13.3 c kg^−1^ for paper and 14.3–21.3 c kg^−1^ for various PAN fiber membranes, respectively. Besides the collectors, the effect of PVDF solutions with different components and concentrations on droplet ejection was also studied (Supplementary Fig. 4 and Supplementary Discussion). Here, we employed the difference (*Δ*) between the actual value and the theoretical droplet threshold of the fluids as an indicator of the capability to burst and generate droplets from the Taylor cone. When using PAN-30 fiber membrane as a collector, the *Δ* achieved the largest value of 10.3 c kg^−1^, indicating the highest formation probability and speed of the droplets during direct electronetting process.

Next, to verify our model predictions, careful SEM observation was conducted to characterize the assembled architectures on various collectors, and the results are shown in Fig. 2e. As predicted by the assembly model, almost exclusively microspheres formed on the flat Al foil due to the lack of microelectric field (Fig. 2e). The use of paper collectors caused a slightly lower charge density of the fluid from Taylor cone (Fig. 2d), and the SEM result matched very well with this prediction: both broken fibers and microspheres were deposited (Fig. 2e). In contrast, the porous collectors, such as Cu mesh and nonwoven fabric, successfully resulted in microelectric fields to facilitate the self-assembly of architectures with 2D network structures. As displayed in Fig. 2e, beaded nanofiber nets and nano-nets self-assembled as expected, and the nonwoven fabric caused better assembly due to its dielectric property that could create a higher microelectric gradient. Meanwhile, obviously more fibrous net deposition could be observed on the Cu mesh collector, further confirming the model prediction that a larger *Δ* (~7.7 c kg^−1^) enhanced the formation probability and speed of the droplets.

Inspired by the successful use of dielectric nonwoven fabric, we further collected the self-assembled architectures using PAN fiber membranes with various pore sizes as substrates (Supplementary Fig. 5), and the typical SEM results are shown in Fig. 2e. The change in pore size led to an obvious structural change in the obtained architectures—broken nano-nets with thinner fiber diameters deposited on the PAN-10 membranes due to excessive stretching that arose from the large pore size (8 μm), while some fibers together with nano-nets formed on the PAN-240 membranes owing to the suppressive charging capability caused by using thicker insulating PAN membranes. Using PAN-30 membranes, desirable nano-nets could be self-assembled by virtue of their optimized combination of dielectric properties and pore size (3 μm) (Fig. 2e). To further confirm the effect of the microelectric field stemming from the pore structure, we prepared a fully compacted PAN-30 fiber membrane using a tablet machine with a pressure of 30 MPa and used it as the collector for direct electronetting. Removal of the pore structure of PAN membranes with almost-unchanged dielectric properties resulted in a dramatic structural change from nano-nets to microspheres, as demonstrated in Fig. 2e. Besides the electric field, the influence of solution system like polymer/additive concentration, molecular weight (*M*_w_) and flow rate on the formation of PVDF nano-nets was also investigated. Almost all the resultant membranes show 2D network structures when using PAN-30 fiber membrane as collectors. With increasing *M*_w_ from 320,000 to 1,100,000, the number density of beads on the fibers significantly decreased due to the enhanced molecular entanglement (Supplementary Fig. 6 and Supplementary Discussion). The flow rate of solution (0.02–0.2 ml h^−1^) obviously affected the deposition efficiency and quality of the nano-net membranes (Supplementary Fig. 7 and Supplementary Discussion).

### Properties of 2D nano-nets

**Fig. 3:**
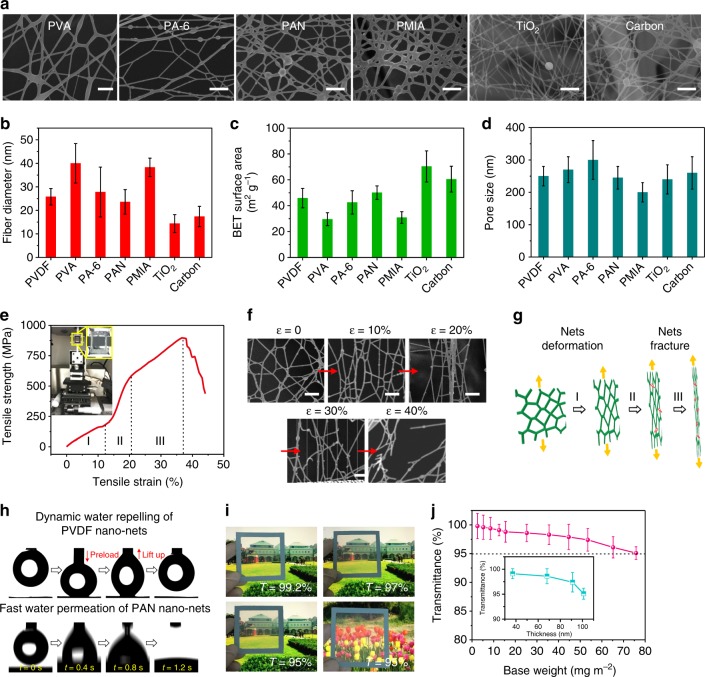
Superior properties of various self-assembled nano-nets. **a** Typical scanning electron microscopy (SEM) images of polyvinyl alcohol (PVA), polyamide-6 (PA-6), polyacrylonitrile (PAN), poly(*m*-phenylene isophthalamide) (PMIA), TiO_2_ and carbon self-assembled nano-nets. Scale bars in (**a**), 300 nm. **b** Fiber diameter, **c** Brunauer–Emmett–Teller (BET) surface area and **d** pore size of the various nano-nets in (**a**). Base weight of the membranes used in (**d**), ~0.05 g m^−2^. **e** Microtensile-strain curve of polyvinylidene fluoride (PVDF) self-assembled nano-nets. The inset image in (**e**) shows a micromechanical tensile tester for a single nanofiber. **f** SEM images of PVDF nano-nets at different tensile elongations (*ε*) during a continuous stretching process. Scale bars in (**f**), 300 nm. **g** Schematic description of the evaluation of the self-assembled nano-nets under continuous tensile deformation. **h** Photographs of dynamic measurements of water adhesion (top) and water permeation (bottom) on the surface of PVDF and PAN nano-nets, respectively. **i** Snapshot images of ultrathin free-standing PVDF self-assembled nano-nets at different transparencies. **j** Transparency values of PVDF nano-nets with different base weights. The inset in (**j**), the corresponding transmittance-thickness curve. Dotted line in (**j**), 95% transmittance. Error bars in (**b**–**d**, **j**) represent s.d.

Because of the simplicity and flexibility of our methodology, great versatility in controlling the material category of the unique 2D nano-nets was possible. Figure 3a shows not only the polymeric nano-nets of polyvinyl alcohol (PVA), polyamide-6 (PA-6), PAN and poly(*m*-phenylene isophthalamide) (PMIA), but also the inorganic nano-nets of TiO_2_ and carbon obtained by combining with a calcination process. The careful analysis of these nano-nets indicated that the polymeric nets showed a fiber diameter range of 20–40 nm, while TiO_2_ and carbon nano-nets achieved even smaller diameters of 14.4 and 17.4 nm, respectively, due to the removal of organic components from their precursor nano-nets, as illustrated in Fig. 3b and Supplementary Fig. 8. Although several pieces of similar networks can be found in holey/lacey carbon or cellulose nanocrystal films after instable polymer/solvent evaporation^26,27^, large-scale fabrication of such nanofibrous networks has never before been reported^46,47^. Here, we can easily fabricate a PVDF nano-net membrane with an area of 55 × 70 cm^2^ (Supplementary Fig. 9 and Supplementary Discussion). Benefiting from their true nanoscale diameters, the nano-nets exhibited greatly enhanced Brunauer–Emmett–Teller (BET)-specific surface areas of 30–72 m^2^ g^−1^ compared to <10 m^2^ g^−1^ of conventional electrospun nanofibers and <15 m^2^ g^−1^ of our previously prepared nanofiber/webs^24^. The respective BET surface areas for these nano-nets are presented in Fig. 3c and Supplementary Fig. 10. This result suggests the promising potential of our nano-nets for use as high-performance sensors, catalyst matrices, optics and photovoltaics^48–51^. As indicated in Fig. 3d and Supplementary Fig. 11, the unique 2D network structures endowed their assembled membranes with extremely small pore sizes of 200–300 nm while maintaining a strikingly low thickness of <100 nm and high porosity of >99.25%. Moreover, the pore distribution of the nano-nets was rather uniform, as illustrated in Supplementary Fig. 12. In dramatic contrast, allowing the control electrospun nanofiber membranes to have a pore size of ~300 nm required a thickness of at least 5 μm, and their porosities were much lower and usually in the range of 60–80%^18,24^. In addition, our direct electronetting process resulted in an obviously enhanced crystallinity and *β*-phase transition of PVDF; the relevant Fourier transform infrared (FT-IR), X-ray diffraction (XRD) and differential scanning calorimetry (DSC) results are shown in Supplementary Fig. 13. In contrast to PVDF powder (crystallinity of 43% and *β*-phase fraction of 56%), the PVDF nano-nets exhibited the crystallinity of 55% and *β*-phase fraction of 85%, due to the synergetic effect of the enhanced electric field poling, uniaxially mechanical stretching and the ion-dipole interaction (Supplementary Discussion), indicating their potential application in high-performance piezoelectric devices and biomedical implants^7,45,52^.

Besides the striking nanostructural features, the nano-nets also exhibited amazing mechanical properties, surface wettability and light transmittance. Using a single-nanofiber tensile tester, we performed dynamic microtensile measurement of the single-layered PVDF nano-nets, and a typical collected curve is illustrated in Fig. 3e. In dramatic contrast to the unrecovered and monotonous deformation of traditional nanofiber membranes, 2D nano-nets can bear a tensile strain as high as 13% without fracture and have a tensile strength of 875 MPa, revealing a typical deformation regime usually observed in mesh-like materials^12,13,39^. To gain insight into this unique deformation, we performed SEM observation of the whole stretching process up to a *ε* of 40%, and the images are shown in Fig. 3f. Nano-nets without tensile strain (*ε* = 0%) typically demonstrated a 2D topological network structure. Subsequently, stretching restricted the nanowires to orient increasingly in a plane parallel to the stretching direction, leading to a dynamic deformation from mesh to slit and producing a slowly increased linear elastic behavior within *ε* of 0–13%. Then, the major nanowires were tightly stretched and almost parallel to one another, thus inducing a rapidly increased and nonlinear deformation behavior within 13–20% *ε*. Beyond 20% *ε*, some junctions between adjacent units were broken first, thus causing the remaining nanowires act as a bundle of nanofibers, showing another slowly increased linear elastic deformation before final structural failure. Based on these results, we further proposed a three-regime break mechanism; the schematic showing the unique gradient deformation involving net deformation and net fracture of 2D nano-nets is presented in Fig. 3g.

The fascinating advantages of the 2D nano-nets can be further demonstrated in terms of their surface wettability and light transmittance. Using a high-speed camera, the dynamic wetting behavior of the nano-nets was monitored. The top images in Fig. 3h depict a 3 μl droplet touching and then detaching from PVDF nano-nets. After full contact with the membrane surface, the droplet was then lifted to leave the surface, showing almost no deformation, thus suggesting superhydrophobicity and low water adhesion. This result can be ascribed to the enhanced hydrophobicity of PVDF nano-nets derived from their nanoscale surface roughness and high porosity according to the Cassie model^19,53^. Evidence of the surface roughness arose from the SEM and atomic force microscopy (AFM) observations of PVDF nano-nets (Supplementary Fig. 14 and Supplementary Discussion). In contrast, the PAN nano-nets exhibited a superior water-adhesion behavior. As illustrated in Fig. 3h (bottom), on PAN nano-net surface, the water droplet spread out quickly (1.2 s), and a negligible contact angle was achieved, indicating superhydrophilicity and high porosity. All these enhanced wettability characteristics can be explained by the selective capillary effect of the nano-nets according to the Laplace theory, which is due to the synergistic effect of the porous nanofibrous structure and material nature. In addition to this superior wettability, the other crucial property for many advanced materials used for electrical devices, bioengineering, optical imaging, etc., light transmittance, was then evaluated. Figure 3i shows photographs of the free-standing PVDF nano-nets with transmittance of ~99.2%, ~97% and ~95%, which can easily allow the sun's rays to shine through, enabling lighting and direct viewing. Both the distant view of buildings and the nearby view of a flower could be seen clearly through a PVDF nano-net membrane with a transmittance of ~95%. No such free-standing nanofiber membranes have ever been reported. The transmittances of our PVDF nano-nets with different base weights or thicknesses are presented in Fig. 3j. Because of the reduced reflection of <30 nm nanowires and the ultrathin thickness of <0.1 μm, the nano-net membranes with base weights of 12.5, 25.3, 53.5 and 75.8 mg m^−2^ achieved 99.1, 98.6, 97.4 and 95.1% transmittance, respectively.

### Functionality of 2D nano-nets

**Fig. 4:**
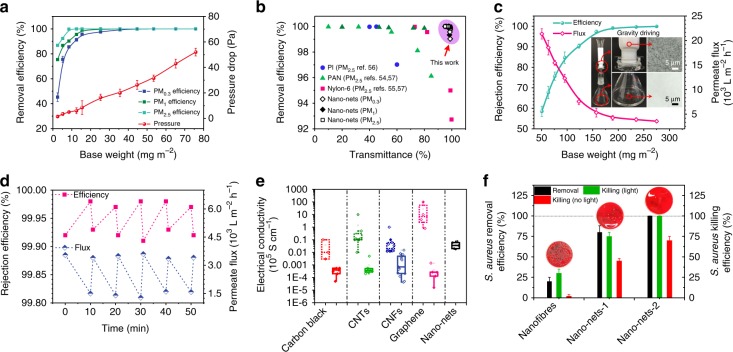
Functionality for applications of air filtration, liquid separation, electric conduction and bioprotective activity. **a** The NaCl PM_0.3_, PM_1_ and PM_2.5_ removal efficiencies and pressure drops of polyvinylidene fluoride (PVDF) nano-net air filters with different base weights. **b** The removal efficiencies of selected nanofiber materials at different transmittance values. **c** Rejection efficiencies and permeate fluxes of polyacrylonitrile (PAN) nano-net filters with different base weights. The inset of (**c**) contains photos showing the process of liquid separation driven by gravity. **d** The cycling separation performance of the PAN nano-net filter. Filter base weight in (**d**), ~275 mg m^−2^. **e** The electrical conductivities of selected carbon materials (carbon black, carbon nanotube (CNT), carbon nanofiber (CNF), graphene and carbon nano-nets) in two forms, bulks and isolated blocks. The solid data boxes correspond to assembled compacts, and the dashed data boxes correspond to their isolated building blocks. The box represents the interquartile range, the center line in the box is the median and the whiskers represent 1.5 times the interquartile range. **f** The removal efficiencies and killing efficiencies against *S. aureus* of TiO_2_ nanofibers and nano-nets. Nano-nets-1, ~50 mg m^−2^. Nano-nets-2, ~120 mg m^−2^. Dotted line in (**f**), 100% efficiency. Error bars in (**a**, **c**, **f**) represent s.d.

Particulate matter (PM) pollution has become a significant burden on global economies and public health^54–57^. Individually, the public tackles this issue using outdoor protective tools, such as facial masks, which are usually of low efficiency, bulky and resistant to airflow, while indoor air quality commonly relies on expensive and energy-intensive air filtration using ventilation systems or central air conditioning. Here, the integrated properties of small pore size, high porosity and high light transmittance allowed the PVDF nano-nets to act as a transparent air filter for windows that used natural passive ventilation to protect indoor air quality. As illustrated in Figs. 3j, 4a, our nano-net filters exhibited excellent removal efficiencies at various transparencies. Filters with a base weight of 15.5 mg m^−2^ showed 95.735% removal for PM_0.3_, 99.028% removal for PM_1_ and 99.972% removal for PM_2.5_ at ~98% transmittance; an increased base weight of 44.7 mg m^−2^ resulted in 99.856% removal for PM_0.3_, 99.993% removal for PM_1_ and 100% removal for PM_2.5_ at ~97% transmittance. In addition, all these filters showed pressure drops of <30 Pa. More strikingly, our PVDF nano-net filter with 75.8 mg m^−2^ could achieve a PM_0.3_ removal efficiency of 99.9992%, which qualified for the standard of ultralow penetration air (ULPA) filters of >99.999%. Meanwhile, at an airflow velocity of 5.33 cm s^−1^, the pressure drop of this ULPA filter was only 0.05% of the atmospheric pressure (52 Pa), which was negligible. Therefore, all these nano-net filters showed robust quality factors for PM_0.3_, PM_1_ and PM_2.5_ removal (Supplementary Fig. 15 and Supplementary Discussion). A comparison between the efficacy of our prepared nano-net filters and the reported records is presented in Fig. 4b. Most existing transparent air filters, such as PAN, PA-6 and polyimide nanofibers, only achieved effective removal for PM_2.5_ or PM_10_ at a limited transmittance of <90%, due to their larger fiber diameters of >100 nm and stacking structures^54–56^. In dramatic contrast, our nano-net filters exhibited a superior removal capacity for smaller and more-penetrating particles (PM_0.3_ and PM_1_) by virtue of their 2D network structure with small pore size (Supplementary Fig. 16a and Supplementary Discussion), while having a lighter weight and thinner thickness, imposing a higher boundary on light transmittance. Besides these advantages, the nano-nets can operate in the transition flow regime due to their high Knudsen number (Kn) of 5.3 (Supplementary Table 4)^17,28^. This result indicates a greatly enhanced slip effect for airflow, leading to a significantly reduced drag force on the nanowires and, thus, extremely low air resistance. In addition, we also studied the long-term recycling operational performance of PVDF nano-net filters for purifying smoke PM_2.5_ and PM_10_ from >500 and >700 μg m^−3^ (extremely hazardous level) to <35 μg m^−3^ (excellent level), as illustrated in Supplementary Fig. 17 and Supplementary Methods. A 25 cm^2^ PVDF nano-net membrane could accomplish a purification process within just 15 min, and this performance remained almost unchanged, even after 15 purification cycles.

Because of the extremely small pore size while maintaining ultrathin thickness combined with the numerous channels derived from the high porosity and superhydrophilicity, effective application of liquid separation by PAN nano-nets was expected. As described in Fig. 4c (inset), using dead-end filtration device, 100 ppm TiO_2_ nanoparticle (diameter 200–400 nm) suspension was directly poured on the prewetted PAN nano-net membrane to test its separation performance. Consequently, this separation process was solely gravity-driven. The water immediately permeated through the nano-net membrane, whereas the particles were captured on the surface. Figure 4c demonstrates the separation performances of PAN nano-net membranes under an external driving pressure of 5 kPa. This separation process is shown in Supplementary Fig. 18. PAN nano-net membranes with base weights of 50.5, 95.3, 185 and 275 mg m^−2^ showed rejection efficiencies of 58.52, 84.69, 99.15 and 99.92% together with permeate fluxes of 21,360 ± 1050, 12,300 ± 820, 4230 ± 480 and 3550 ± 275 L m^−2^ h^−1^, respectively. These separation fluxes were almost one order of magnitude higher than those of conventional membranes with similar rejection properties, such as commercial microfiltration and ultrafiltration membranes^19,58,59^. In addition, using a cycling separation test (Supplementary Methods), the reusability of our PAN nano-net membranes was further examined; the obtained performance for 50 min cycling is shown in Fig. 4d. The result indicates that the permeation flux decreased (from ~3500 to 1500 L m^−2^ h^−1^) while the rejection efficacy increased (from 99.92% to 99.98%) over time, which could be attributed to the generation of a filter cake on the membrane surface. Therefore, this process is a typical surface filtration due to the physical sieving capacity originating from the extremely small pores of 2D nano-nets. Strikingly, by simple cleaning with water, the permeation flux was completely recovered, a feature that has rarely been found in other porous separation materials with such rejection efficacy^19^.

Despite the exceptional electrical conductivity of the isolated building blocks of different carbon nanomaterials, perhaps the biggest challenge to be faced is how to manipulate these blocks to effectively bring their remarkable electrical properties onto the macroscopic level^60–64^. To achieve this aim, various strategies have been used, including cross-linking, compacting, alignment, etc., but their resultant bulk conductivities were still rather low^65,66^. Here, our findings further indicate that a facile carbonization of the as-prepared PAN nano-nets can yield conductive carbon nano-nets, and one layer of such nets even can act as a macroscopic graphene sheet. The carbon nano-nets still retained the unique network structure after pyrolysis at 850 °C (Fig. 3a); thus, robust conductivity was expected. As shown in Fig. 4e, most existing highly conductive carbon materials (like carbon black, carbon nanotubes (CNTs), carbon nanofibers (CNFs) and graphene) in bulk form show dramatically decreased conductivity compared with their isolated blocks. For instance, even when metal cross-links were used between graphene sheets instead of physical bonds, conductivities in the range of 0.1–20 s cm^−1^ were reached; however, they were several orders of magnitude below those of graphene sheets (10^5^–10^6^ s cm^−1^)^60,61,65^. In contrast, our multilayer carbon nano-nets that functioned as a macroscopic material could easily achieve electrical conductivities ranging from 180 to 750 s cm^−1^, having five times higher values than those of CNF bulks (1–150 s cm^−1^) and CNT bulks (20–80 s cm^−1^); the detailed comparison between most of existing electrospun PAN-based CNFs and nano-nets is shown in Supplementary Fig. 19 and Supplementary Discussion. This result could be attributed to the integrated and continuous 2D network structure of carbon nano-nets, which led to a continuously and steadily conductive path or network that was difficult to achieve for isolated carbon building blocks, even when scaled up into mat or paper forms. This advantage supports the use of carbon nano-nets as a stand-alone material or filler for conductive composites in supercapacitors, batteries, sensors, electromagnetic interference shielding, electrostatic discharge protection, and so on^61,64,67^.

Emerging infectious diseases (EIDs) have caused serious public health issues^68–70^. Most existing personal protective equipment (PPE), such as bioprotective suits and medical gloves, used to prevent EID transmission and infections is usually devoid of antimicrobial activity^70^. Because of their robust removal efficacy and antibacterial activity, the incorporation of TiO_2_ nano-nets as a biocidal surface protective layer on PPE was promising. To evaluate the antibacterial activity of our TiO_2_ nano-nets, we challenged the membrane surface with typical *Staphylococcus* *aureus*, and the results are illustrated in Fig. 4f. For the *S. aureus* removal assay, the control (electrospun TiO_2_ nanofibers) and TiO_2_ nano-net membranes with a size of 2.5 cm^2^ were fed with 5 ml ~1.0 × 10^8^ colony-forming units (CFUs) ml^−1^ of *S. aureus* inoculum, and both the feed and filtrate inoculum were assessed by agar plate counting. Due to their unique network structure and extremely small pore size, the TiO_2_ nano-nets with base weights of 50 and 120 mg m^−2^ had significantly enhanced removal efficiencies of 80% and 99.999% compared to the TiO_2_ nanofibers (~20%). The SEM observation indicates that *S. aureus* particles were firmly captured on the nano-net surface (Supplementary Fig. 16b and Supplementary Discussion). Meanwhile, a contact-killing assay of these samples with and without light irradiation was also conducted. The control nanofiber samples displayed a low killing efficiency of 30%, even after light irradiation, and a negligible efficiency (2%) without light. In contrast, the TiO_2_ nano-nets showed effective killing of *S. aureus*, achieving various contact-killing levels: 75% killing with light and 45% killing without light at 50 mg m^−2^ base weight and 99.99% killing with light and 71% killing without light at 120 mg m^−2^ base weight. Notably, the *S. aureus* killed by anatase TiO_2_ having robust antibacterial capacity (Supplementary Fig. 20) were continuously attached and filtered by the nano-nets, avoiding the re-pollution of the pathogens (Supplementary Fig. 16c). This result indicates TiO_2_ nano-nets have similar antimicrobial efficacy and 1/10 weight of cutting-edge antibacterial nanofiber membranes^70^. The nanoscale diameter (14.4 nm), surface filtration function and ultrathin property of TiO_2_ nano-nets were responsible for their robust *S. aureus* killing performance: the ultrathin thickness admitted sufficient light to support the photoinduced antibacterial activity on the enlarged surface area, facilitating the rapid killing of *S. aureus* captured on the net surface. Our findings show that TiO_2_ nano-nets can serve as an effective biocidal layer that not only intercepts but also kills pathogens, suggesting intriguing potential applications in bioprotective PPE against increasing EID threats.

## Discussion

The successful synthesis of self-assembled nano-nets using direct electronetting provides a robust methodology to explore the assembly of nanofibers and their applications in a nanoarchitectured, 2D porous, pseudo-3D macroscopic form. Here, some selected polymers, carbon- and TiO_2_-based nano-nets, served as model systems for a proof of concept. In considering the ease of optimization of precursor solutions and the diversity of receiving collectors, our findings provide a versatile platform for designing new types of nano-nets for use in various applications. For example, biocompatible materials (like polycaprolactone and poly(lactic acid)), could be processed into 2D nano-nets to achieve a more sophisticated mimic of the extracellular matrix, thus enhancing the exchange of nutrients and metabolism and directing cell interactions, migration and morphogenesis. Furthermore, by combination with a calcination process, a variety of inorganic nano-nets (such as those containing silica and inorganic metal oxide) can also be expected and used for high-temperature filters, photovoltaic and photocatalytic devices and electrical energy storage. In addition, other functions, such as catalytic activity, magnetic properties and adsorption capability, could be readily endowed with nano-net composites by simply incorporating functional fillers (for instance, precious metal nanoparticles and metal-organic framework nanosheets).

In summary, the work described here presents a one-step strategy for the scalable synthesis of 2D, self-assembled nano-nets using a unique direct electronetting technology. Pure nanoarchitectured fibrous networks with nanoscale fiber diameters (10–40 nm) and 2D topological network structures, avoiding the formation of electrospun nanofibers (0.2–2 μm), have been assembled into membranes with tunable pore structures and desirable assembly structures. With the integrated superproperties of their nanostructural features, robust mechanical strength, enhanced surface wettability, high transparency and unique functionality in terms of air filtration, liquid separation, electric conduction and bioprotective activity, we can expect that such exceptional methodology for creating nano-nets will offer ample opportunities for a wide range of applications in individual protection, environmental governance, tissue engineering, public health, electronics and energy.

## Methods

### Fabrication of self-assembled 2D nano-nets

The precursor solutions used in this work were prepared by dissolving polymers (PVDF, PVA, PA-6, PAN, PMIA, titanium isopropoxide and polyvinylpyrrolidone) in compatible solvents (*N,N*-dimethylacetamider (DMAc), H_2_O, formic acid (HCOOH), *N,N*-dimethylformamide (DMF), acetic acid (CH_3_COOH), and ethanol) with optimized additives (lithium chloride (LiCl) and sodium chloride (NaCl)). The detailed solution compositions and concentrations used for preparing different self-assembled nano-nets are shown in Supplementary Table 5. All resultant nano-net membranes were fabricated using a DXES-V spinning machine (SOF Nanotechnology Co., Ltd., China). The precursor solutions were loaded into three 10-ml syringes with 20-G metal needles and pumped out at a designed feed rate ranging from 0.02, 0.05, 0.1 to 0.2 ml h^−1^. A high voltage of 25 kV was supplied to the needle tips to form levitating clusters of highly charged droplets. Designed collector substrates including aluminum foil, polyethylene nonwoven fabric, resin-impregnated cellulose paper, copper mesh and PAN fiber membranes were employed to wrap the grounded metal roller (rotating speed of 40 rpm) to collect the resultant architectures, i.e., microspheres, beaded nanofiber membranes and nano-net membranes. Notably, the spinning duration of PAN fiber membranes formed as substrates from 13 wt% PAN solutions was adjusted to 5, 10, 20, 30, 60, 90, 120 and 240 min to study the effect of the dielectric properties and pore structure of collectors on the architectures obtained using the direct electronetting technique. Meanwhile, concaves with different widths on polymethyl methacrylate films were created by laser ablation using a high-precision laser etching system (Fermi Laser Technology Co., Ltd., China). The direct electronetting was performed in a programmable laboratory with controllable temperatures of 20–25 °C and humidities of 40–50%. In addition, an annealing process at 600 °C in air for 1 h with a heating rate of 2 °C min^−1^ was employed to prepare TiO_2_ nano-nets from the resultant hybrid membranes, while a stabilization process in air at 280 °C for 2 h and a subsequent carbonization process in a 99.999% nitrogen flow at 850 °C for 2 h with a heating rate of 2 °C min^−1^ was used to fabricate carbon nano-nets from our resultant PAN nano-net membranes.

### Characterization

The charge density of the liquids ejected from Taylor cone during the direct electronetting process was measured according to the typical mesh target method using a Fluke F15B+ multimeter, and the properties of the precursor solutions, such as viscosity, conductivity and surface tension, were also tested systematically, as depicted in our previous work^25^. The microscopic architecture and chemical structure of the self-assembled nanofibrous materials were characterized by field-emission SEM (Hitachi S-4800), AFM (NT-MDT Ntegra, monocrystal silicon tip, tapping mode), FT-IR spectroscopy (Nicolet 8700), XRD (D/Max-2550 PC) and DSC (Netzsch DSC 204 F1). The base weight and thickness of the nano-net membranes were determined using a Mettler Toledo Micro balance (AT-20, readability of 2 μg) and a Labthink high-precision thickness gauge (CHY-C2) respectively. The pore structures of the nano-net membranes were measured using a CFP-1100AI capillary flow porometer (Porous Materials Inc., USA), while the specific surface areas of the membranes were characterized by N_2_ adsorption–desorption isotherms using an ASAP2020 surface area analyzer (Micromeritics Co., Norcross, GA). The micromechanical property data of the PVDF nano-nets were collected using a FSF001.1 single-nanofiber tensile tester with a precision of 0.0001 cN (Suzhou Intel-Rising Technology Co., Ltd., China); a frame was used to collect the testing samples of a bundle of nano-nets by placing it on the collector for 1–2 s during the electronetting process, and the samples were confirmed to consist of just one layer before testing using an Olympus BH2-UMA microscope equipped with an IS Capture image program. The relative permittivity of the collector substrates, such as nonwoven fabric, paper and PAN fiber membranes, was examined using a KRM9530 high-resistance meter (Kruoma Technology Ltd., China). The water contact angle (3 μl) measurement of PVDF and PAN nano-net membranes was performed using a SL200B contact angle goniometer (Kino Industry Co., Ltd., USA). The transmittance spectrum of the free-standing PVDF nano-net filters was weighted using an Ideaoptics IS-30–6-R integrating sphere attached to the PG 2000+ optic spectrum from 400 to 800 nm to obtain the average transmittance (see details in Supplementary Methods).

The NaCl PM removal efficiency and pressure drop of the self-assembled nano-net air filters were evaluated using a LZC-K filter tester (Huada Filter Technology Co., Ltd., China), and the details of the filtration measurement are presented in Supplementary Methods. The TiO_2_ nanoparticle suspension used for testing the liquid separation performance of the PAN nano-net membranes was characterized and confirmed by optical microscopy (Olympus VHS3000, Japan). The concentrations of the feed TiO_2_ suspension (100 ppm) and the filtrate were measured using the PG 2000+ UV-visible spectroscopy system, and the rejection efficiency was calculated using the equation $\left( {1 - C_{\mathrm{{p}}}/C_{\mathrm{{f}}}} \right) \times 100$, in which *C*_p_ and *C*_f_ represent the TiO_2_ concentration in the filtrate and feed suspension, respectively. Their cycling separation performance was evaluated after reverse cleaning using deionized water (Supplementary Methods). The electrical resistivity was determined with a ST-2258C multifunction digital four-point probe apparatus (Suzhou Jingge Electronic Co., Ltd., China). A clinical isolate of *S. aureus* (SA 1004) with a concentration of ~1.0 × 10^8^ CFU ml^−1^ was used to evaluate the bioprotective activity of the TiO_2_ nano-net membranes, and the detailed measurement is shown in Supplementary Methods.

## Supplementary information

Supplementary Information

## Data Availability

The experimental data that support the findings of this study are available from the corresponding author upon reasonable request.
