# Publisher Correction: Direct electronetting of high-performance membranes based on self-assembled 2D nanoarchitectured networks

**DOI:** 10.1038/s41467-020-14864-2

**Published:** 2020-02-20

**Authors:** Shichao Zhang, Hui Liu, Ning Tang, Jianlong Ge, Jianyong Yu, Bin Ding

**Affiliations:** 1 State Key Laboratory for Modification of Chemical Fibers and Polymer Materials, College of Textiles, Donghua University, Shanghai, 201620 China; 2 Innovation Center for Textile Science and Technology, Donghua University, Shanghai, 200051 China

**Keywords:** Synthesis and processing, Two-dimensional materials, Polymers

Correction to: *Nature Communications* 10.1038/s41467-019-09444-y, published online 29 March 2019.

The original version of this Article incorrectly omitted the received/accepted dates of Received: 11 October 2018; Accepted: 11 March 2019. This has now been corrected in the PDF and HTML versions of the Article.

