# Supplementary material for: Direct electronetting of high-performance membranes based on self-assembled 2D nanoarchitectured networks
Source: Nat Commun. 2019 Mar 29;10:1458. doi: 10.1038/s41467-019-09444-y (PMC6441005; doi:10.1038/s41467-019-09444-y)
Supplement: Supplementary file 1 — Supplementary Information [file 41467_2019_9444_MOESM1_ESM.pdf]

# Direct Electronetting of High-Performance Membranes Based on Self-Assembled 2D Nanoarchitectured Networks

Zhang *et al.*

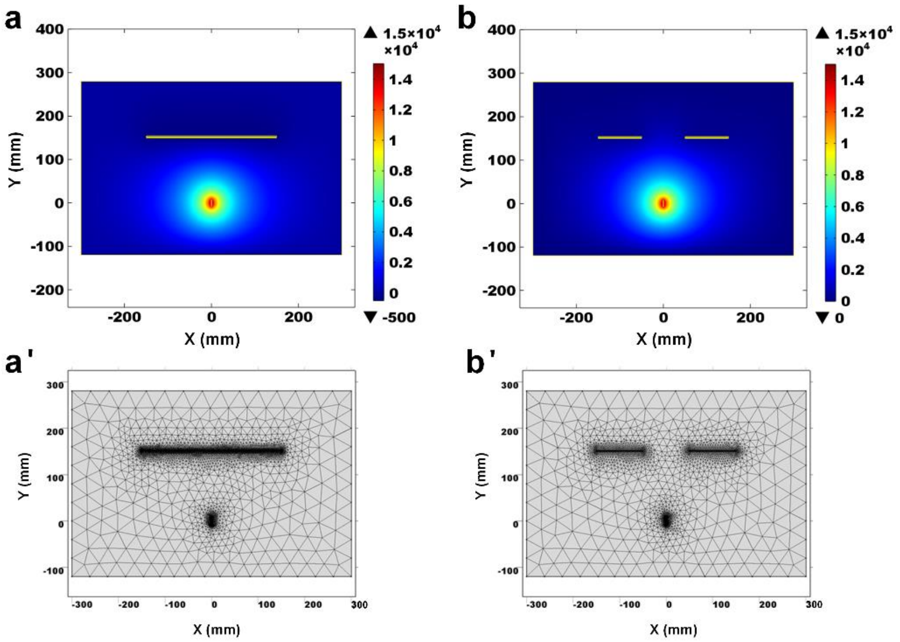


**Supplementary Figure 1 Electric field simulation of the direct electronetting process.** Potential distribution of the electric field during the direct electronetting process using (a) a flat dielectric collector (i.e., paper) and (b) a concave conductive collector (i.e., Cu mesh). (a') and (b') are the finite element axisymmetric meshing of electric fields (a) and (b), respectively.

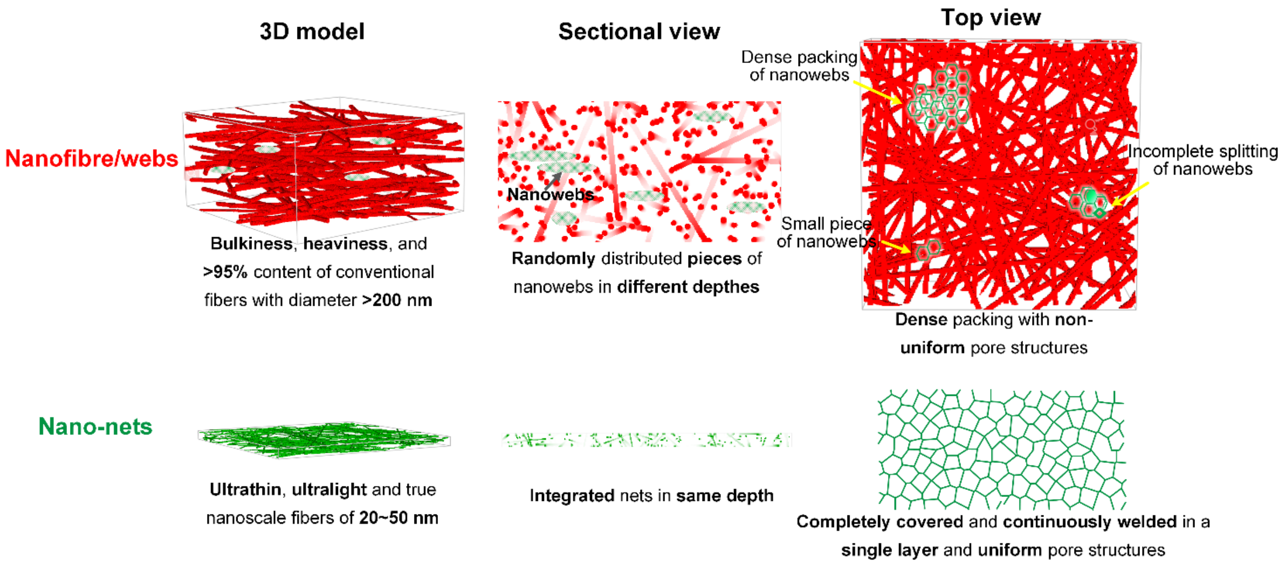


**Supplementary Figure 2 Structural models of nanofibre/webs and nano-nets.** Schematic models showing the essential differences in structures between our previously prepared nanofibre/webs and the 2D self-assembled nano-nets obtained in this work.

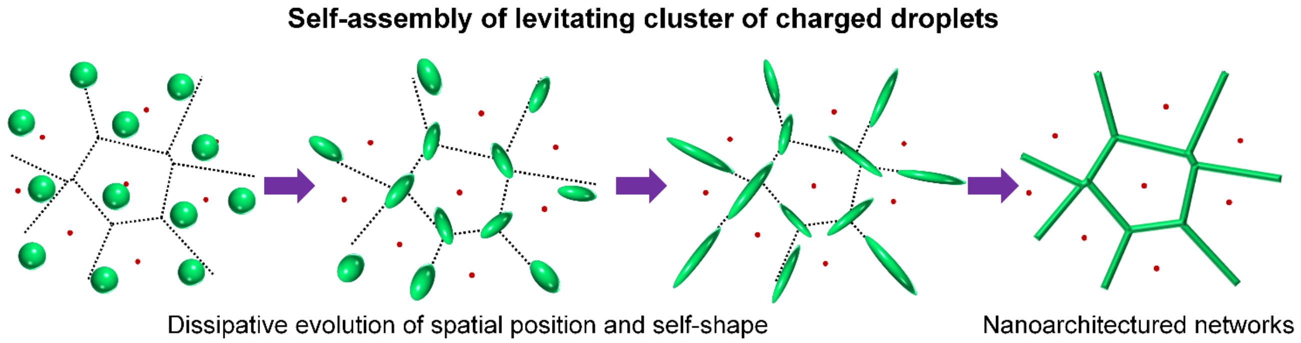


**Supplementary Figure 3 Possible formation process for 2D nano-nets.** Schematic illustrating the self-deformation and self-assembly of the levitating cluster of charged droplets.

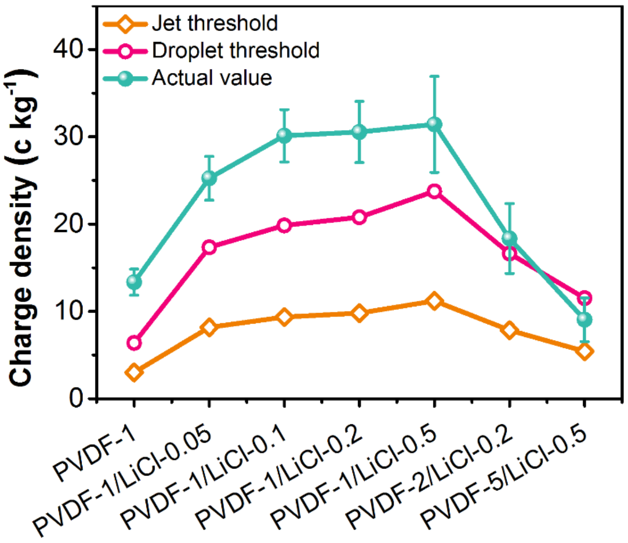


**Supplementary Figure 4 Charge densities of the fluids from various PVDF/LiCl solutions during direct electronetting.** Charge density (charge-to-mass ratio) of the fluids ejected from various PVDF/LiCl solutions with different PVDF concentrations and LiCl concentrations during the direct electronetting process. PVDF-X/LiCl-Y, X and Y are the PVDF and LiCl concentrations, respectively (wt%). Error bars represent s.d.

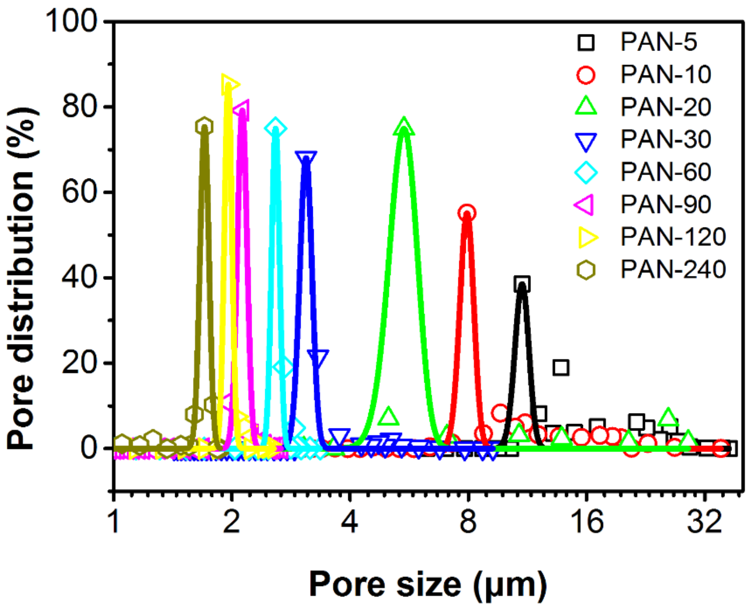


**Supplementary Figure 5 Pore structures of PAN fibre membranes.** Pore size distribution of electrospun PAN fibre membranes as collectors for direct electronetting with various spinning durations. PAN-X, X is the spinning duration (min).

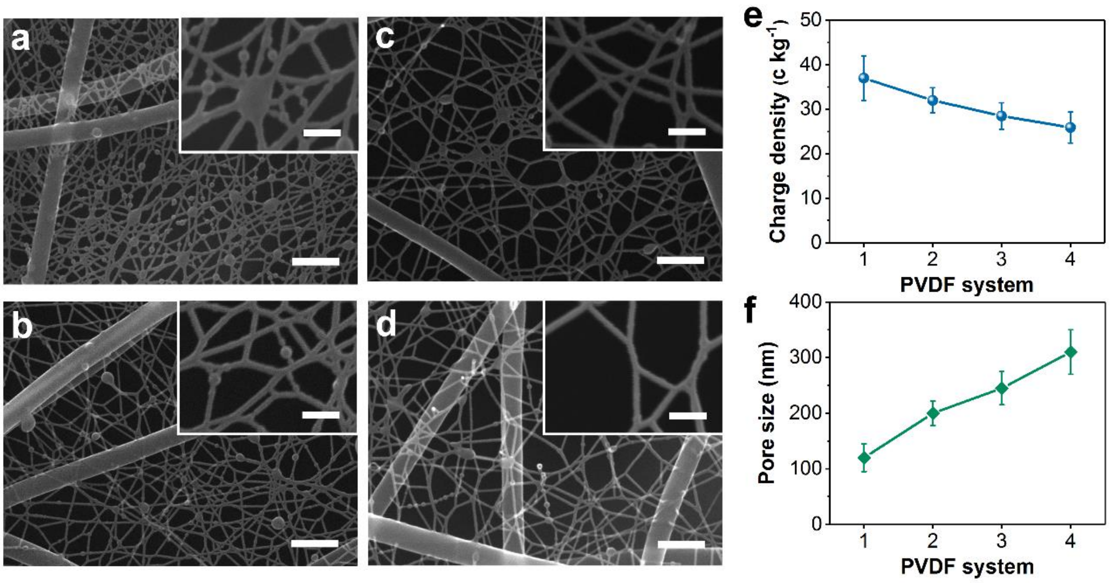


**Supplementary Figure 6 The effect of molecular weight on the formation of PVDF nano-nets.** Typical SEM images of the nano-nets formed from PVDF solutions with different molecular weights $M_w$ of (a) 320,000, (b) 570,000, (c) 680,000 and (d) 1,100,000. Scale bars in (a-d), 1 μm. Scale bars in the insets of (a-d), 300 nm. (e) Charge density of the liquids and (f) pore size of the membranes formed from the corresponding solutions. Error bars in (**e**) and (**f**) represent s.d.

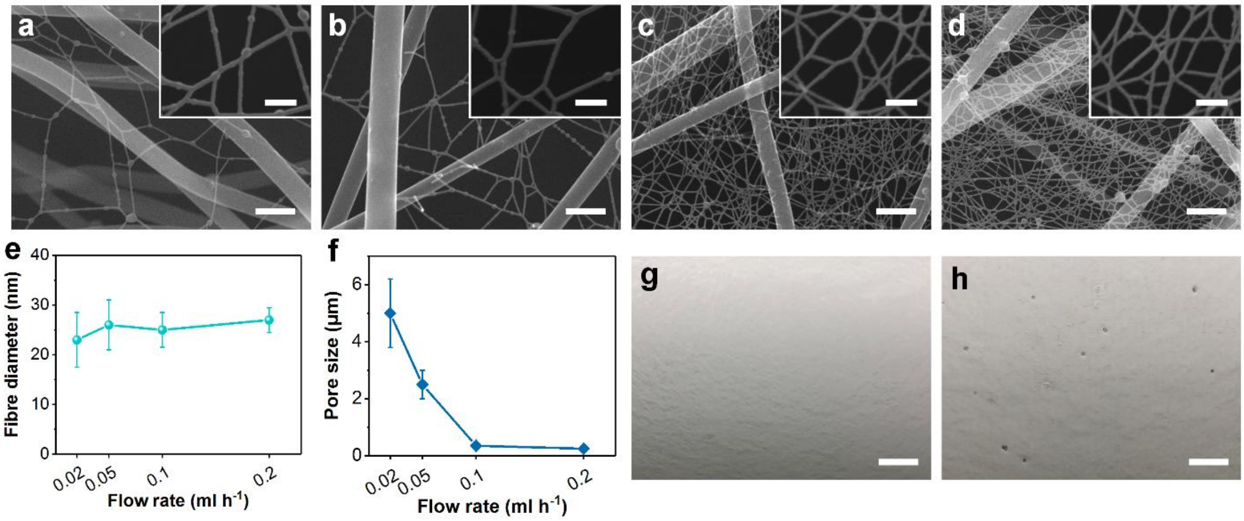


**Supplementary Figure 7 The effect of flow rate on the structure of PVDF nano-nets.** Typical SEM images of the self-assembled nano-nets formed using different flow rates of (a) 0.02, (b) 0.05, (c) 0.1 and (d) 0.2 ml $h^{-1}$. Scale bars in (a-d), 1 μm. Scale bars in the insets of (a-d), 300 nm. (e) Fibre diameter and (f) pore size of the membranes formed using the corresponding flow rates. Photographs of the resulted nano-net membranes fabricated using flow rates of (g) 0.1 and (h) 0.2 ml $h^{-1}$. Base weight of the membranes used in (g) and (h), ~0.5 g $m^{-2}$. Error bars in (**e**) and (**f**) represent s.d.

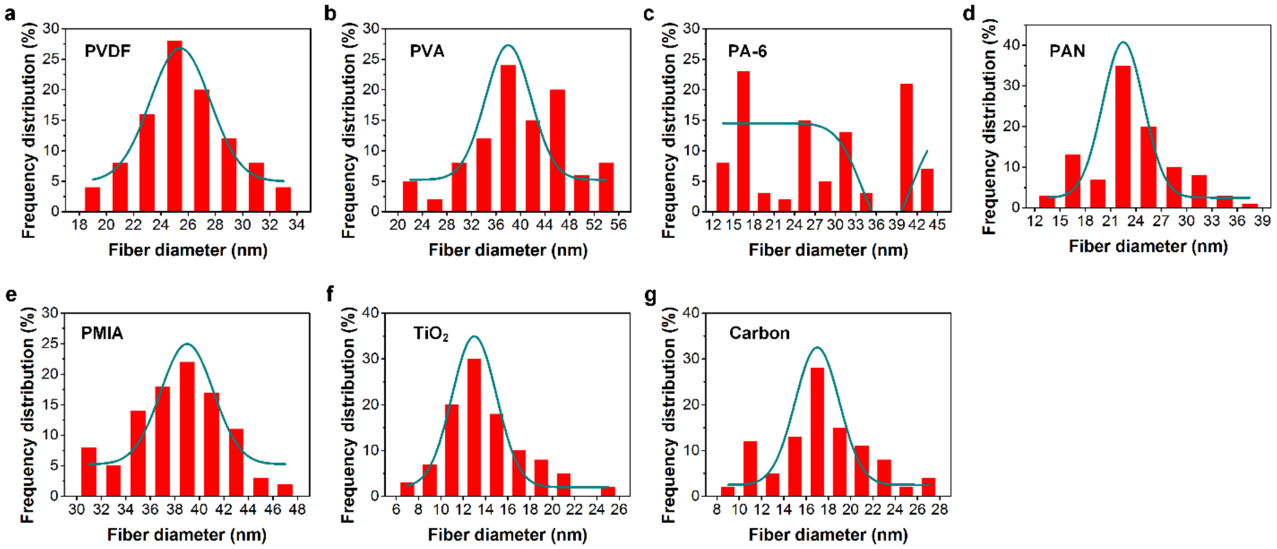


**Supplementary Figure 8 Fibre diameters of nano-nets from different polymers, metallic oxide and carbon.** Histogram showing the fibre diameter distribution of (a) PVDF, (b) PVA, (c) PA-6, (d) PAN, (e) PMIA, (f) $TiO_2$ and (g) carbon nano-nets.

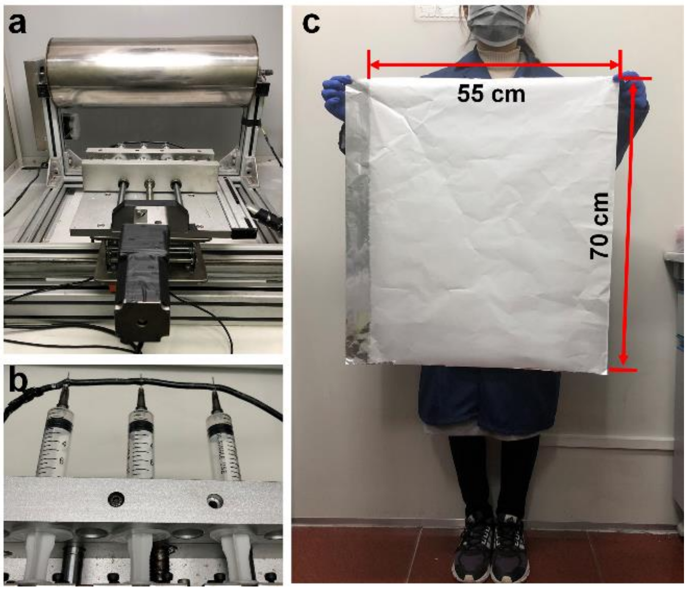


**Supplementary Figure 9 Large-area fabrication of the nano-net membranes.** Photographs showing (a) DXES-V spinning lab equipment, (b) 3 syringes loaded on the filling pump and (c) PVDF nano-net membrane with area of 55 × 70 $cm^2$.

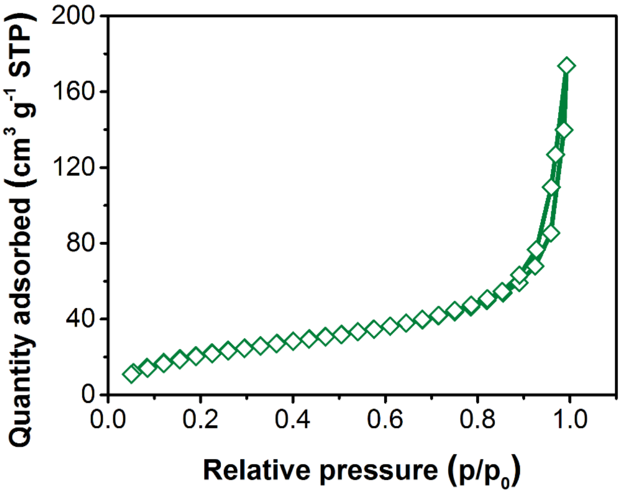


**Supplementary Figure 10 BET analysis of PVDF nano-nets.** $N_2$ adsorption-desorption isotherm of PVDF nano-net membranes.

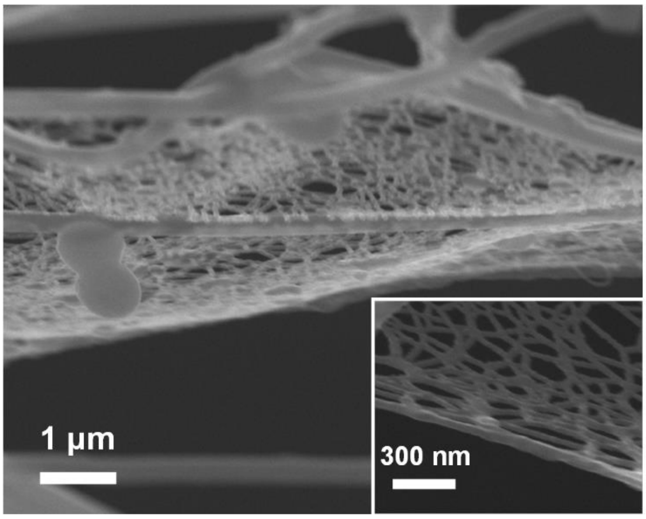


**Supplementary Figure 11 Cross section of PVDF nano-nets.** FE-SEM image of the cross section of PVDF nano-nets supported by PAN fibres. Filter base weight, 0.12 mg $m^{-2}$.

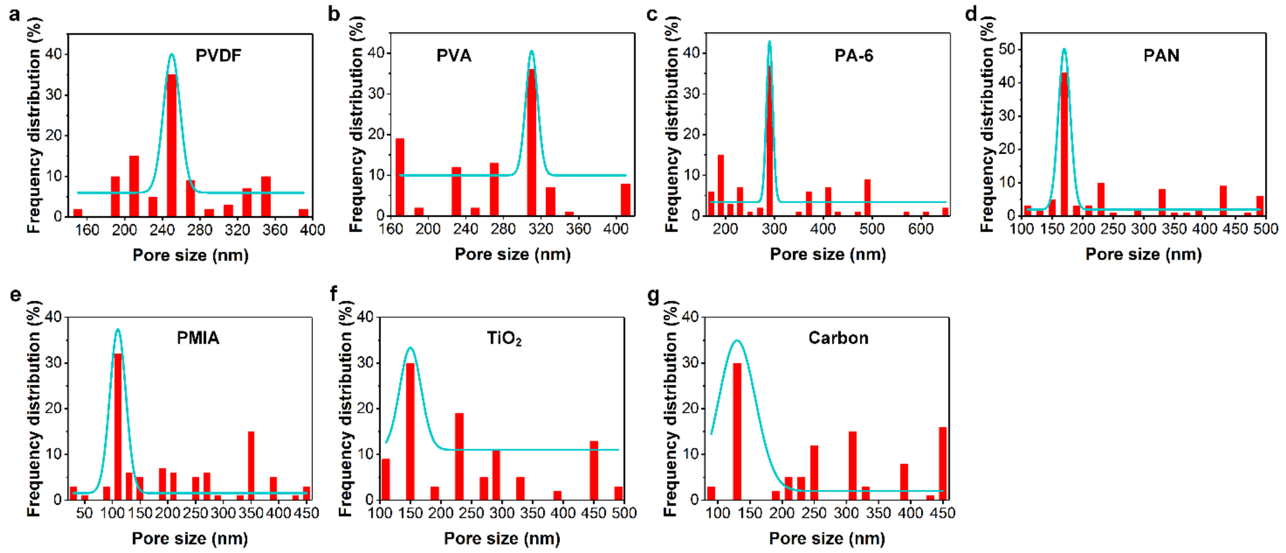


**Supplementary Figure 12 Pore sizes of nano-nets from different polymers, metallic oxide and carbon.** Histogram showing the pore size distribution of (a) PVDF, (b) PVA, (c) PA-6, (d) PAN, (e) PMIA, (f) $TiO_2$ and (g) carbon nano-nets.

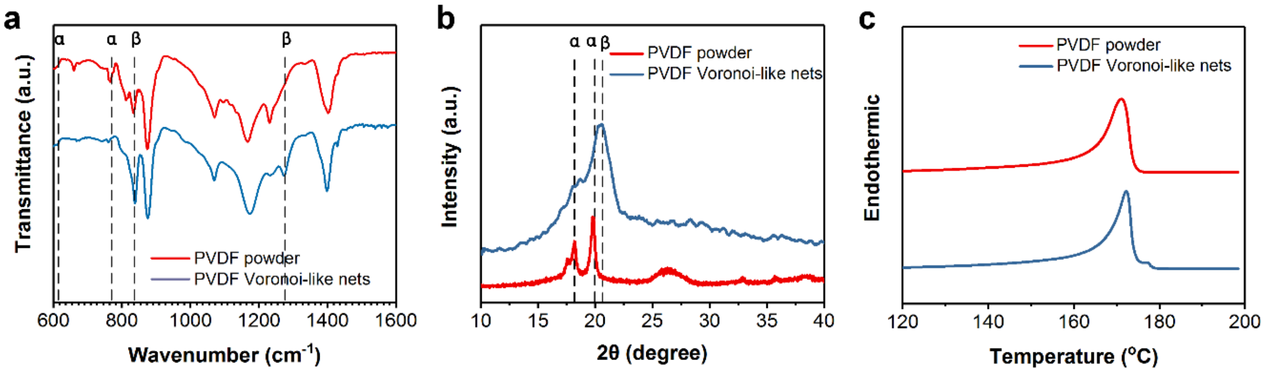


**Supplementary Figure 13 Crystallinity and crystal phase of the PVDF nano-nets.** (a) FTIR spectra, (b) XRD patterns and (c) DSC thermograms of PVDF nano-net membrane and PVDF powder.

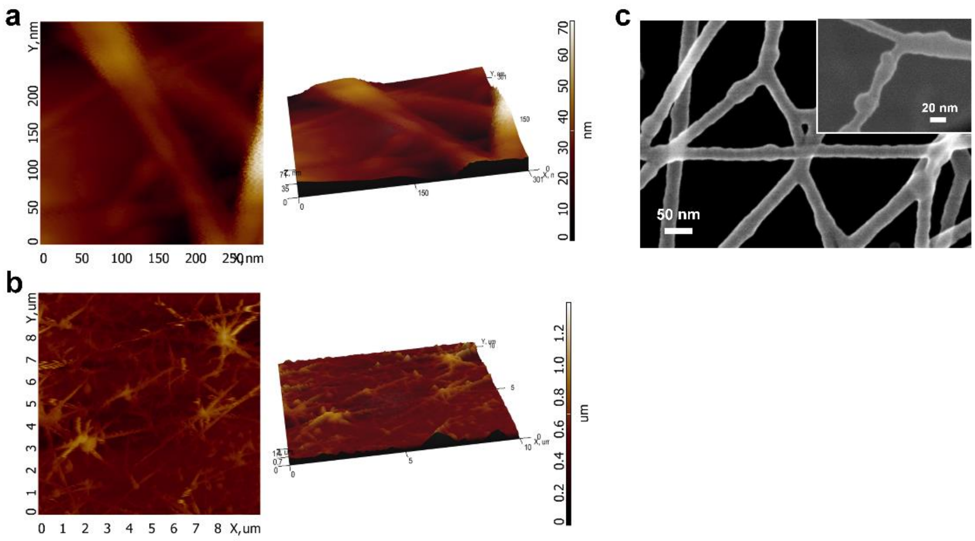


**Supplementary Figure 14 Topography of nanofibres in 2D nano-nets.** AFM images (left, 2D; right, 3D) of topography with area of (a) 300 × 300 nm$^2$ and (b) 10 × 10 μm$^2$ of the PVDF nano-nets. (c) high-resolution FE-SEM images of the PVDF nano-nets.

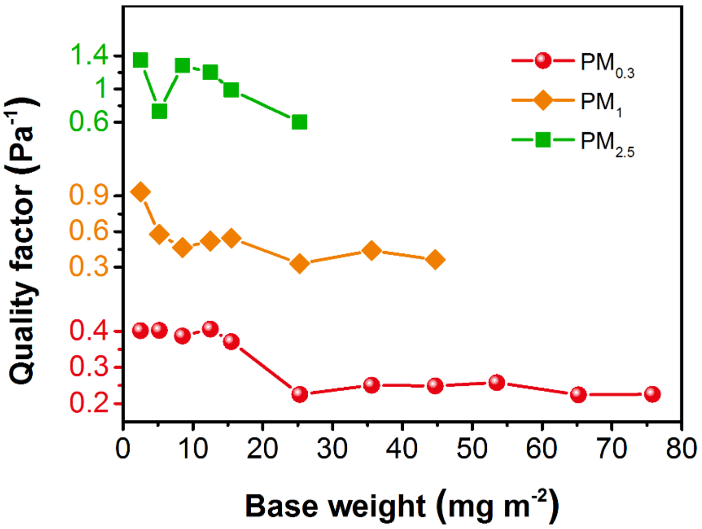


**Supplementary Figure 15 Quality of PVDF nano-net air filters.** Quality factor of PVDF nano-net air filters with various base weights for NaCl $PM_{0.3}$, $PM_1$ and $PM_{2.5}$ capture.

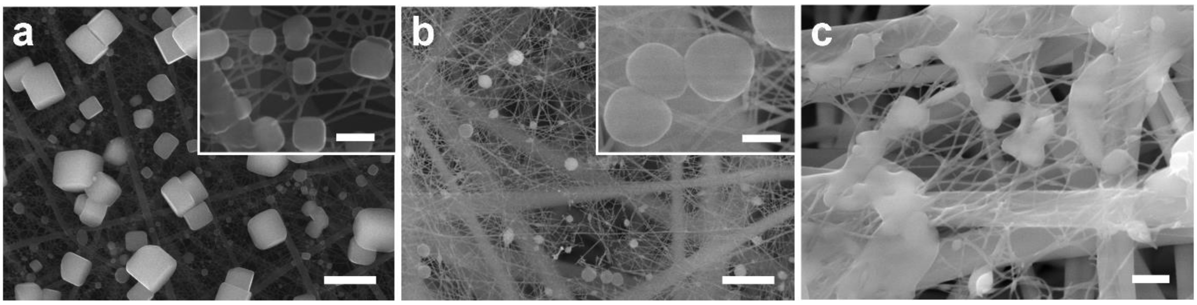


**Supplementary Figure 16 Removal of PM and bacterium using nano-nets.** SEM images of (a) PVDF nano-nets after NaCl PM filtration, and $TiO_2$ nano-nets after bacterium removal (b) pre and (c) post killing process. Scale bars in (a and b), 3 μm. Scale bars in the insets of (a and b), 500 nm. (c) Scale bar in (c), 1 μm.

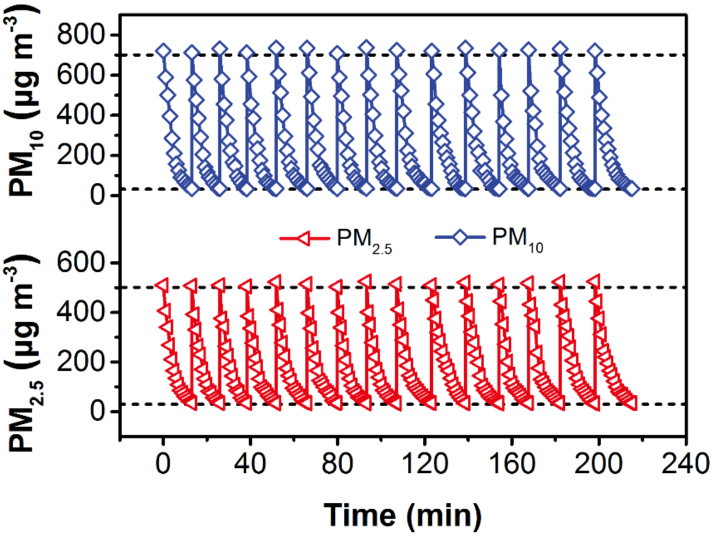


**Supplementary Figure 17 Long-term purification of PVDF nano-nets for PM pollution.** Long-term recycling performance in removing $PM_{10}$ (from >700 to <35 μg $m^{-3}$) and $PM_{2.5}$ (from >500 to <35 μg $m^{-3}$). Airflow velocity, 5.33 cm $s^{-1}$. Filter base weight, ~80 mg $m^{-2}$.

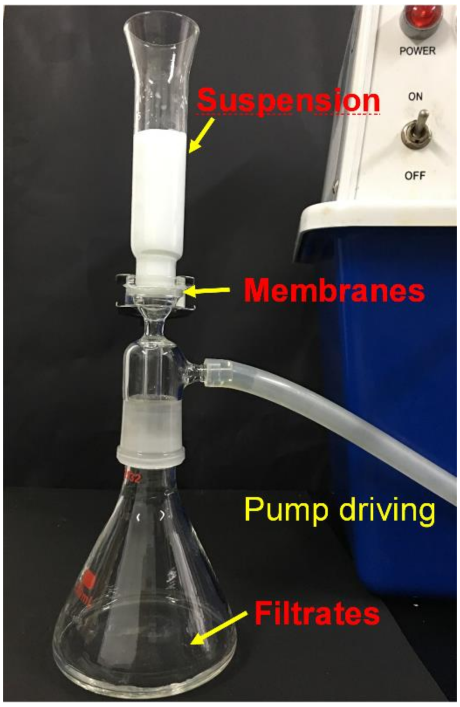


**Supplementary Figure 18 Liquid separation process of PAN nano-nets under the driving of external pressure.** Digital photo showing the separation process of a $TiO_2$ nanoparticle suspension using a PAN nano-net membrane under the driving of external pressure. Filter base weight, ~200 mg $m^{-2}$.

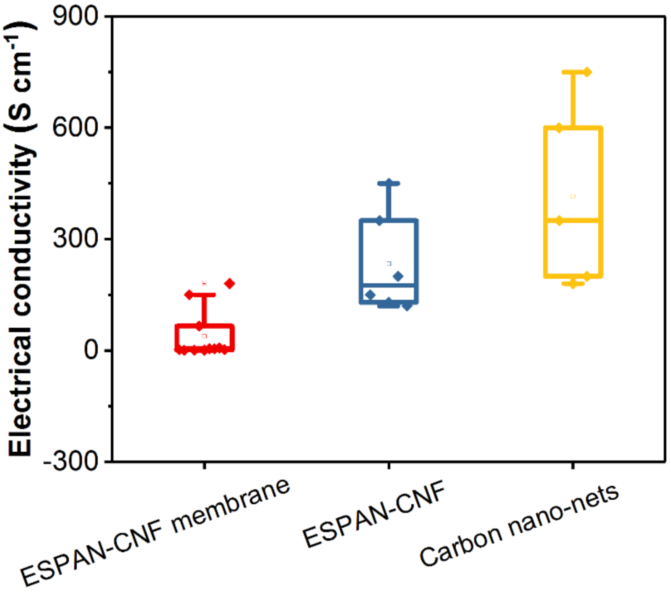


**Supplementary Figure 19 Comparison of electrical conductivity of electrospun PAN based carbon nanofibres and nano-nets.** The electrical conductivities of electrospun PAN based carbon nanofibres (membranes and single fibres) and carbon nano-nets. The box represents the interquartile range, the horizontal line in the box is the median, and the whiskers represent 1.5 times the interquartile range.

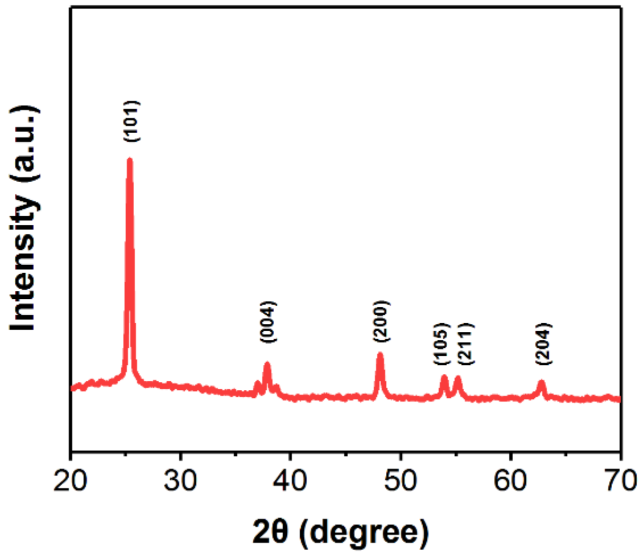


**Supplementary Figure 20 Crystal phase of the $TiO_2$ nano-nets.** XRD pattern of $TiO_2$ nano-net membrane.

**Supplementary Table 1 Model and parameters for simulation of the electrostatic field.**

| Model | Needle | Collector | Shielding net | Air medium |
|---|---|---|---|---|
| **Dimension (mm)** | Width: 0.5<br>Height: 15 | Width: 300<br>Height: 1 | Width: 600<br>Height: 400 | Width: 600<br>Height: 400 |
| **Coordinate (mm)** | X: 0<br>Y: 0 | X: –150<br>Y: +150 | X: 0<br>Y: 80 | X: 0<br>Y: 80 |
| **Relative Dielectric Constant** | 2 | - | 5 | 1 |
| **Voltage (V)** | 15000 | 0 | 0 | - |

**Supplementary Table 2 Parameters for fabricating materials used for SEM observation.**

| Sample | Polymer | Polymer concentration /wt% | Solvent | LiCl concentration /wt% | Voltage /kV | Distance /cm | Temperature /ºC | Humidity /% | Substrate |
|---|---|---|---|---|---|---|---|---|---|
| **Fig. 2a** | PVDF | 1 | DMAc | 0.1 | 25 | 15 | 20−25 | 45 ± 5 | Concave Al foil |
| **Fig. 2b** | PVDF | 1 | DMAc | 0.1 | 25 | 15 | 20−25 | 45 ± 5 | Concave PMMA film |
| **Image 1 in Fig. 2e** | PVDF | 1 | DMAc | 0.1 | 25 | 15 | 20−25 | 45 ± 5 | Al foil |
| **Image 2 in Fig. 2e** | PVDF | 1 | DMAc | 0.1 | 25 | 15 | 20−25 | 45 ± 5 | Nonporous paper |
| **Image 3 in Fig. 2e** | PVDF | 1 | DMAc | 0.1 | 25 | 15 | 20−25 | 45 ± 5 | Cu mesh |
| **Image 4 in Fig. 2e** | PVDF | 1 | DMAc | 0.1 | 25 | 15 | 20−25 | 45 ± 5 | Nonwoven fabric |
| **Image 5 in Fig. 2e** | PVDF | 1 | DMAc | 0.1 | 25 | 15 | 20−25 | 45 ± 5 | PAN-10 membrane |
| **Image 6 in Fig. 2e** | PVDF | 1 | DMAc | 0.1 | 25 | 15 | 20−25 | 45 ± 5 | PAN-30 membrane |
| **Image 7 in Fig. 2e** | PVDF | 1 | DMAc | 0.1 | 25 | 15 | 20−25 | 45 ± 5 | PAN-240 membrane |
| **Image 8 in Fig. 2e** | PVDF | 1 | DMAc | 0.1 | 25 | 15 | 20−25 | 45 ± 5 | Compacted PAN-30 membrane |

**Supplementary Table 3 Effects of the collectors and polymer systems on the charge states of fluids during direct electronetting.**

| Substrate | PAN-5 | PAN-10 | PAN-20 | PAN-30 | PAN-60 | PAN-90 | PAN-120 | PAN-240 | Non-woven | Paper | Al foil | Cu mesh |
|---|---|---|---|---|---|---|---|---|---|---|---|---|
| **Solution** | 1 wt% PVDF, 0.1 wt% LiCl | | | | | | | | | | | |
| **ε (F m$^{-1}$)** | 3.345E-10 | | | | | | | | | | | |
| **$\gamma$ (mN m$^{-1}$)** | 36.32 | | | | | | | | | | | |
| **$\rho$ (kg m$^{-3}$)** | 946.3 | | | | | | | | | | | |
| **$\eta$ (cps)** | 8.6 | | | | | | | | | | | |
| **$K$ (μs cm$^{-1}$)** | 1240 | | | | | | | | | | | |
| **$\delta$** | 1.138 | 1.187 | 1.232 | 1.318 | 1.565 | 1.779 | 2.109 | 2.521 | 1.572 | 2.925 | - | - |
| **$J_c$** | 10.07 | 9.86 | 9.68 | 9.36 | 8.59 | 8.05 | 7.39 | 6.76 | 8.57 | 6.28 | 10.74 | 9.35 |
| **$D_c$** | 21.36 | 20.91 | 20.53 | 19.85 | 18.21 | 17.08 | 15.69 | 14.35 | 18.17 | 13.32 | 22.78 | 19.85 |
| **Actual value** | 25.6 | 26.75 | 28.44 | 30.12 | 29.59 | 24.64 | 17.32 | 11.9 | 18.12 | 10.1 | 22.05 | 27.48 |

| Substrate | PAN-30 | | | | | |
|---|---|---|---|---|---|---|
| **PVDF (wt%)** | 1 | 1 | 1 | 1 | 2 | 5 |
| **LiCl (wt%)** | 0 | 0.05 | 0.2 | 0.5 | 0.2 | 0.5 |
| **$\gamma$ (mN m$^{-1}$)** | 35.17 | 36.3 | 37.39 | 37.52 | 37.24 | 36.8 |
| **$\rho$ (kg m$^{-3}$)** | 945.2 | 945.8 | 947.5 | 950.9 | 956.1 | 985.4 |
| **$\eta$ (cps)** | 9 | 9 | 9.33 | 9.67 | 15 | 37 |
| **$K$ (μs cm$^{-1}$)** | 3.1 | 662 | 1716 | 3970 | 1897 | 3442 |
| **$\delta$** | 1.318 | | | | | |
| **$J_c$** | 3.01 | 8.18 | 9.8 | 11.2 | 7.84 | 5.43 |
| **$D_c$** | 6.39 | 17.34 | 20.79 | 23.76 | 16.64 | 11.52 |
| **Actual value** | 13.35 | 25.25 | 30.55 | 31.42 | 18.35 | 9.05 |

**Supplementary Table 4 Knudsen numbers and flow regimes dependent on different ranges of fibre diameter under normal conditions.**

| Knudsen number ($Kn$) | Flow regimes | Fibre diameter (d) |
|---|---|---|
| $Kn < 0.001$ | Continuum flow regime | d > 132 μm |
| $0.001 < Kn < 0.25$ | Slip flow regime | 528 nm < d < 132 μm |
| $0.25 < Kn < 10$ | Transition flow regime | 13.2 nm < d < 528 nm |
| $Kn > 10$ | Free molecular flow regime | d < 13.2 nm |

**Supplementary Table 5 Detailed compositions and concentrations of precursor solutions for the fabrication of various nano-nets.**

| **Nano-nets** | PVDF | | | | | | |
|---|---|---|---|---|---|---|---|
| Polymer/material | PVDF chips | | | | | | |
| Solvent | DMAc | | | | | | |
| Additive | LiCl | | | | | | |
| Polymer concentration /wt% | 1 | 1 | 1 | 1 | 1 | 2 | 5 |
| LiCl concentration /wt% | 0 | 0.05 | 0.1 | 0.2 | 0.5 | 0.2 | 0.5 |

| **Nano-nets** | PVA | PA-6 | PAN | PMIA | $TiO_2$ | Carbon |
|---|---|---|---|---|---|---|
| Polymer/material | PVA chips | PA-6 chips | PAN powders | Teijinconex ® PMIA staple fibers | Titanium isopropoxide, polyvinylpyrrolidone | PAN chips |
| Solvent | $H_2O$ | HCOOH | DMF | DMAc | $CH_3COOH$ /ethanol (1/2) | DMF |
| Additive | NaCl | -- | LiCl | LiCl | -- | LiCl |
| Polymer concentration /wt% | 1.5 | 2 | 3 | 1 | PVP | 3 |
| Additive concentration /wt% | 0.1 | -- | 0.1 | 0.1 | -- | 0.1 |

## Supplementary Note 1

| **Nomenclature** | | | |
|---|---|---|---|
| $e$ | charge of fluid | $l$ | length of hypothetical cylindrical jet |
| $m$ | mass of fluid | $F_\gamma$ | hydrostatic pressure of fluid |
| $e/m$ | charge density of fluid | $F_e$ | Coulomb repulsion of fluid |
| $\varepsilon$ | ambient permittivity | $E$ | electric field intensity |
| $\gamma$ | surface tension of fluid | $V$ | electric potential of fluid |
| $\rho$ | density of fluid | $W$ | electric energy of fluid |
| $D$ | diameter of charged fluid/Taylor cone apex | $dW$ | change in electric energy of fluid |
| $R$ | radius of charged fluid/Taylor cone apex | $Q$ | volumetric flow rate |
| $\delta$ | correction factor | $J_c$ | jet threshold |
| $x$ | distance between studied location and center of charged fluid | $D_c$ | droplet threshold |
| $\eta$ | viscosity of fluid | $F_E$ | electrostatic force |
| $K$ | conductivity of fluid | $F_f$ | air drag |

**Ejection models of the Taylor cone apex.** In this work, we chose the diameter of the Taylor cone apex at the maximum curvature as the characteristic diameter of the ejected fluid, and it could be calculated by the following parametric equation[1-3].

$$D = 1.46Q^{0.44}\varepsilon^{0.12}\eta^{0.32}K^{-0.12}\gamma^{-0.32} \qquad (1)$$

Due to the tiny weight, the effect of gravity on the dynamic evolution of the studied liquid is negligible; therefore, their ejection modes are mainly driven by the competition between Coulombic repulsion $F_e$ and hydrostatic pressure $F_\gamma$ (refs 4-6). When $F_e > F_\gamma$, two distinct ejection modes could occur: jet mode and droplet mode. For the jet mode, a cylindrical fluid (i.e., jet) is assumed to eject from the

Taylor cone, which is a common phenomenon during the electrospinning process. The $F_\gamma$ caused by the surface tension of the fluid can be calculated using the following formula:

$$F_\gamma = {}^{\gamma}/_{R} \tag{2}$$

For simplicity, the charged fluid and external electric field were studied as a closed and independent system. Considering that the conductivity of the receiving substrate would greatly influence the electric field distribution, the correction factor ($\delta$) developed from the relative permittivities of the substrates was introduced to evaluate the contribution of the electric potential, concentrating on enhancing the charge density of the liquids compared with conventional electrospinning. According to classical electromagnetism, the electric field intensity $E$ at the extension cord of the axis of the cylindrical jet can be expressed as:

$$E = {}^{\delta e}/_{4\pi\varepsilon x^2} \tag{3}$$

Then, we can deduce the electric potential $V$ and electric energy $W$ at the end of the cylindrical jet:

$$V = {}^{\delta e}/_{4\pi\varepsilon l} \tag{4}$$

$$W = {}^{\delta e^2}/_{8\pi\varepsilon l} \tag{5}$$

When the charge of the jet remains unchanged while its length increases from $l$ to $l + dl$, the change in electric energy $dW$ can be expressed as:

$$dW = \frac{-\delta e^2}{8\pi\varepsilon l^2} dl \tag{6}$$

Based on the work-energy theorem, we can also obtain the $dW$ through calculating the work done by Coulomb repulsion:

$$dW = -\pi R^2 F_e dl \tag{7}$$

Then, the Coulomb repulsion $F_e$ can be deduced on the basis of Supplementary Equations (6) and (7):

$$F_e = {}^{\delta e^2}\!/_{8\varepsilon\pi^2 R^2 l^2} \tag{8}$$

In addition, the mass of the studied cylindrical fluid *m* is:

$$m = \frac{1}{4}\rho\pi D^2 l \tag{9}$$

Therefore, the jet threshold can be deduced on the basis of Supplementary Equations (1), (2), (8) and (9):

$$J_c = \sqrt{{}^{64\varepsilon\gamma}\!/_{\delta\rho^2 D^3}}$$

While for droplet mode, the $F_\gamma$ of the droplet can be calculated by:

$$F_\gamma = {}^{2\gamma}\!/_{R} \tag{10}$$

Then, the electric field intensity *E* can be deduced according to Gauss's law:

$$E = {}^{\delta e}\!/_{4\pi\varepsilon x^2} \tag{11}$$

And the electric energy (*W*) at the surface of the charged droplet and its change (*dW*) when the radius is increased from *R* to *R* + *dR* can be obtained:

$$W = \int_0^e \int_R^\infty E dx\, de = \frac{-e^2}{8\pi\varepsilon R^2} dR \tag{12}$$

$$dW = \frac{-\delta e^2}{8\pi\varepsilon R^2} dR \tag{13}$$

Based on the work-energy theorem, the *dW* can also be calculated:

$$dW = -4\pi R^2 F_e dR \tag{14}$$

Thus,

$$F_e = {}^{\delta e^2}/_{32\varepsilon\pi^2 R^4} \tag{15}$$

Additionally, the mass of the charged droplet $m$ is:

$$m = \frac{1}{6}\rho\pi D^3 \tag{16}$$

Similarly, the droplet threshold can be deduced on the basis of Supplementary Equations (1), (10), (15) and (16):

$$D_c = \sqrt{{}^{288\varepsilon\gamma}/_{\delta\rho^2 D^3}}$$

# Supplementary Discussion

## The possible formation process for 2D self-assembled nano-nets.

Mother Nature's legacy not only makes Voronoi cells visible in nature, as in dragonfly wings, turtle carapaces and honeycombs, but shows ordered dynamic evolution phenomena as well. To decrease the entropy similarly to Rayleigh-Bénard convection, many dissipative structures form, for instance, naturally growing snow crystals and levitating clusters of water droplets in clouds or on a heated water surface. Here, our obtained nano-nets may be regarded as another similar case. By revealing the deformation and self-assembly of the charged droplets during direct electronetting, we proposed a possible formation process for 2D nanofibrous networks. The schematic showing this process is illustrated in Supplementary Fig. 3. During direct electronetting, abundant charged droplets were ejected from Taylor cone and flew with a high speed toward the collector, which could be regarded as a levitating cluster within a stable and continuous process. The levitating droplets repelled with each other and powered by the electric field gradient, underwent rapid self-assembly of their spatial position based on dissipative effect to achieve an energy/material minimum state. Meanwhile, rapid stretching deformation occurred in the droplets flying toward the topographically structured collectors due to the electrostatic force stemming from the differential microelectric fields (Fig. 1d). This deformation and deposition driven by microelectric fields were a common phenomenon for patterning of electrospun fibres, and have been widely used to tailor nanofibre structures for some special applications. Further stretching and solvent evaporation might result in the connection of adjacent deformed droplets to reduce the surface energy of the whole droplet cluster. Thus, nanofibre membranes with ideal or weighted 2D networks (i.e., nano-nets or beaded nanofibre nets) were obtained after evaporation of the solvent phase and solidification of the polymer phase.

**The effect of solution systems on the formation of 2D self-assembled nano-nets.**

In addition to the collectors, the solution system was also an important factor that greatly influenced the formation of the self-assembled architectures; therefore, we also established the numerical prediction diagram for various PVDF/LiCl solutions with different components and concentrations for direct electronetting, as illustrated in Supplementary Fig. 4. The increase of the LiCl concentration while maintaining an unchanged PVDF concentration (1 wt%) caused the gradual increase of the jet and droplet thresholds. For instance, with increasing LiCl concentration from 0, 0.05, 0.1, 0.2 to 0.5 wt%, the droplet threshold of these systems increased from 6.38, 17.3, 19.8, 20.8 to 23.8 c $kg^{-1}$, and their actual charge density increased, as well, from 13.4, 25.3, 30.1, 30.6 to 31.4 c $kg^{-1}$ due to the significantly increased conductivity and viscosity of the used solutions. When using the solution with 1 wt% PVDF and 0.1 wt% LiCl, the difference (Δ) between the actual value and theoretical droplet threshold of the fluids achieved the highest value of 10.3 c $kg^{-1}$, indicating the highest probability for the formation of droplets during the direct electronetting process. Further increasing the PVDF and LiCl concentration at the same weight ratio (PVDF/LiCl of 10/1) led to reduction of the jet and droplet threshold: the droplet thresholds of liquids from a 2 wt% PVDF/0.2 wt% LiCl solution and a 5 wt% PVDF/0.5 wt% LiCl solution were 16.6 and 11.5 c $kg^{-1}$ respectively. This increased concentration also greatly reduced their actual charge density, which could be attributed to the suppression of molecular movement and decreased surface tension (Supplementary Table 3 and Supplementary Note 1).

In addition to solution concentration, the effect of molecular weight on the formation of 2D self-assembled nano-nets was also investigated. Typical SEM images of the nano-nets formed from PVDF solutions with different molecular weights are illustrated in Supplementary Fig. 6a-d. Besides the molecular weight, these four membranes are fabricated using the same solution concentration, processing conditions and deposition duration. Obviously, almost all the membranes exhibited 2D network structures. With increasing the molecular weight, the deposition density of the nanofibrous

networks decreased significantly, resulting in an obviously increased pore size of the membranes. As shown in Supplementary Fig. 6f, an increase in the molecular weight from 320,000 to 1,100,000 caused a sharp increase in the pore size of the resulted membranes from 120 to 310 nm. This result can be ascribed to the change of charge density of the liquid, which greatly affects the formation efficiency of the droplets during direct electronetting process. As illustrated in Supplementary Fig. 6e, the PVDF solutions with lower molecular weight are usually much easier to be charged and would carry more charges. For example, the charge density of liquid from PVDF solution with molecular weight of 320,000 is ~37 c $kg^{-1}$, while that of the solution with molecular weight of 1,100,000 is ~26 c $kg^{-1}$. And this increased charge density would enhance the formation of charged droplets and then lead to more deposition of the nanofibrous networks in the same electronetting duration. Meanwhile, from the SEM images we can see that, the number density of beads on the fibres obviously decreased with increasing the PVDF molecular weight. This phenomenon could be attributed to the better molecular entanglement of higher molecular weight, which can facilitate the formation of uniform jets during electrohydrodynamic process.

As observed in Supplementary Fig. 7a-d, with increasing the flow rate of the solution from 0.02 to 0.2 ml $h^{-1}$ during the direct electronetting, the deposition amount of the nano-net membranes was greatly enhanced within the same electronetting duration. All the membranes show 2D network structures, and have a similar fibre diameter in the range of 20-30 nm, as shown in Supplementary Fig. 7e. This result may be ascribed to that the PVDF solution with same component and concentration (even though different flow rates) can be fully charged, and then form nanofibres with similar diameters after a similar stretching and solidification process in the same electric field. While, due to the increased deposition amounts, the membranes fabricated using higher flow rates exhibited an obvious decrease of their pore sizes; the obtained results are shown in Supplementary Fig. 7f. For instance, with increasing the flow rate from 0.02 to 0.1 ml $h^{-1}$, the pore size of the nano-net membrane decreased from 5 to 0.35 μm. While, further increasing the flow rate to 0.2 ml $h^{-1}$ caused a slight

decrease of the pore size of the membranes (0.3 μm). We attributed this result to the superfluous flow rate of 0.2 ml $h^{-1}$, which usually caused abound drops to fall from the needle, rather than to form nanofibres on the collector. More interestingly, using the high flow rate of 0.2 ml $h^{-1}$, some droplets could not have enough time to deform and solidify, and would cause macroscopical defects (pores) on the membrane, as shown in Supplementary Fig. 7g and h. And these defects typically deteriorate the mechanical property and performance of the membranes, especially for filtration and separation applications. Therefore, to achieve a rapid fabrication speed while maintaining the quality of the membranes, in this work we chose a flow rate of the solution of 0.1 ml $h^{-1}$ to fabricate the nano-net samples for various applications, like air filtration, liquid separation, electric conductivity and bioprective activity.

**Large-area fabrication of 2D self-assembled nano-nets.**

With respect to the large-area fabrication, even industrial process, two important issues should be carefully considered: raw materials and fabrication process. Here, raw materials are commercialized with low cost. Because of the simplicity of the one-step self-assembly process of our methodology and the facile availability of the designed precursor solutions and collectors, we think large-scale fabrication of 2D nano-nets was possible. Actually, using our lab equipment (DXES-V spinning machine, SOF Nanotechnology Co., Ltd., China), we could easily obtain an uniform nano-net membranes with an area of 55 × 70 $cm^2$ using 3 syringes and a collecting roller (length of 60 cm and diameter of 24 cm), as shown in Supplementary Fig. 9. And we have confidence in the fabrication of nano-net membranes with larger area using more syringes and larger collector.

**Crystallinity of the PVDF nano-net membranes.**

PVDF, as one of the semicrystalline thermoplastic polymers, has been found to have a strong piezo, pyro, and ferroelectric property, and its piezoelectric coefficient is almost 10 times larger than other polymers[7]. PVDF has five crystalline phases: non-polar $\alpha$-phase and $\varepsilon$-phase, as well as polar $\beta$-

phase, $\delta$-phase, and $\gamma$-phase, in which the $\beta$-phase can lead to the highest permanent dipole and then improve piezoelectric property. The electrical poling and stretching processes usually can align the dipoles in the crystalline PVDF structures to facilitate the formation of $\beta$-phase. Here, we also investigated the influence of electronetting process on the PVDF crystallinity. The FTIR spectra of the PVDF powder and the nano-net membrane are shown in Supplementary Fig. 13a. Obviously, the bands at 840 and 1278 $cm^{-1}$ which correspond to the $\beta$ phase can be observed for the nano-net membrane. We further calculated the relative content of $\alpha$ and $\beta$ polymorphs using the following equation: $C(\beta) = A_\beta / (1.26A_\alpha + A_\beta)$, where $A_\alpha$ and $A_\beta$ are the transmittance bands at 763 and 840 $cm^{-1}$, respectively. The fraction of $\beta$ phase of PVDF nano-net membrane and PVDF powder is 85% and 56%, respectively, indicating a transition from $\alpha$ to $\beta$ phase can be achieved by direct electronetting. Supplementary Fig. 13b shows the XRD patterns of the PVDF powder and the nano-net membrane. The peaks at around $2\theta = 17.6^o$ and $2\theta = 20.1^o$, which correspond to 100 and 110 crystal planes of the $\alpha$-phase, are obvious for the PVDF powder. In contrast, a very strong diffraction peak at $2\theta = 20.6^o$ corresponding to 110 and 200 reflections of the $\beta$-phase crystal, can be clearly observed for the nano-net membrane, further confirming the transition from $\alpha$-phase to $\beta$-phase after electronetting. The DSC melting traces of the PVDF powder and the nano-net membrane are presented in Supplementary Fig. 13c. The melting temperature ($T_m$) of PVDF powder (171.1ºC) is lower than nano-nets (173.8 ºC). Furthermore, the degree of crystallinity ($\Delta X_c$) of each sample was determined from the DSC curves using equation: $\Delta X_c = \Delta H / (x_\alpha \Delta H_\alpha + x_\beta \Delta H_\beta)$, where $\Delta H$ is the melting enthalpy of the sample under consideration, the $x_\alpha$ and $x_\beta$ indicate the $\alpha$- and $\beta$- phase content while $\Delta H_\alpha$ and $\Delta H_\beta$ are the melting enthalpies of a 100% crystalline sample in the $\alpha$- and $\beta$- phases in the sample. The value of $\Delta H_\alpha$ is 93.07 J $g^{-1}$ and $\Delta H_\beta$ is 103.4 J $g^{-1}$, respectively. The obtained result showed that the PVDF nano-nets had higher crystallinity (55%) than that (43%) of PVDF powder. We attributed the enhanced formation of polar $\beta$ phase in PVDF nano-nets to the following two pivotal aspects: (i) The dramatically increased solution

conductivity (due to the addition of ionic salt LiCl) caused remarkable enhancement of the electric field poling and uniaxially mechanical stretching during direct electronetting process. The dipoles of PVDF chains were, therefore, aligned entirely along the direction of the electric field, and such strengthened dipole orientation promoted the transition of $\alpha$-phase into $\beta$-phase. (ii) PVDF/DMAc/LiCl, intermolecular interaction, further facilitated the $\beta$-crystalline phase formation. DMAc/LiCl solution can usually generate $[DMAc+Li]^+$ macrocation and Cl- anion. The positive $CH_2$ dipoles ($\delta$+) and negative $CF_2$ dipoles ($\delta$-) in the PVDF chains could form the ion-dipole interaction with the Cl- anion and the $[DMAc+Li]^+$ macrocation, which promoted the transition to the $\beta$-phase crystals.

**Surface roughness of the PVDF nano-nets.**

The material hydrophobicity is usually due to hierarchical micro/nanostructures. In this work, we have performed AFM observation of the PVDF nano-nets, the result is shown in Supplementary Fig. 14a and b. The AFM measurement was performed using an NT-MDT Ntegra AFM equipped with polysilicon lever with a monocrystal silicon tip (tip curvature radius <10 nm). Cantilever type is length of 94 μm, width of 34 μm, thickness of 1.85 μm, force constant of 12 N $m^{-1}$, resonant frequency of 235 kHz. And the AFM mode used during the experiment is tapping mode. Here, the used PVDF nano-net samples for AFM testing were collected using $SiO_2$ wafer, which is a kind of dielectric film without porous structure (similar as the substrate of paper used in this work). Due to lack of the microelectric field, we only got the broken nanofibres and microspheres on the $SiO_2$ wafer; and the fibres typically can not tightly attach on the wafer. Therefore, during the AFM testing, the tapping of the tip easily caused the moving of the tiny nanofibre with diameter of ~40 nm, leading to an image which is not clear enough to check the roughness on the single nanofibre surface (Supplementary Fig. 14a). While, obvious roughness on a larger scanning area can be found on the $SiO_2$ wafer, which is due to the deposition of the PVDF nanofibres (Supplementary Fig. 14b). To further check the surface roughness on single nanofibre in the nano-nets, we have also performed the high-resolution FE-SEM imaging.

As illustrated in Supplementary Fig. 14c, dense and obvious bulges or wrinkles formed on the surface of PVDF nanofibres, which can be attributed to the fast phase separation and solidification of the charged liquid during direct electronetting[8,9]. These nanostructures on fibre surface were beneficial for enhancing the surface roughness and creating hierarchical structure, and then resulted in an enhancement effect for the hydrophobicity of PVDF nano-nets.

**Quality factor of 2D nano-net air filters.**

In general, the quality factor (*QF*) is an important trade-off indicator to evaluate the filtration capacity of air filters based on their removal efficiency and air resistance and can be defined by the formula: $QF = -\ln(1 - \eta)/\Delta p$, in which the $\eta$ is the removal efficiency and the $\Delta p$ is the pressure drop[10-13]. From Supplementary Fig. 15, we can find that, upon increasing the base weight of the nano-net filters, their *QF* for the removal of PM with various sizes gradually decreased. This result could be due to the increased packing density of the nano-net filters with higher base weights. However, all *QF* values of the nano-net air filters maintained high levels for $PM_{0.3}$, $PM_1$ and $PM_{2.5}$ removal when compared with commercial air filters or electrospun nanofibre membranes. For instance, the *QF* values were in the ranges of 0.22−0.4 $Pa^{-1}$ for $PM_{0.3}$ removal, 0.3−0.9 $Pa^{-1}$ for $PM_1$ removal, and 0.6−1.3 $Pa^{-1}$ for $PM_{2.5}$ removal. Commercial materials with macrosized diameters (for example, melt-blown fibres and glass fibres), although they can achieve a similar removal efficiency by virtue of the electret effect and the unlimited increase of basis weight (>100 g $m^{-2}$, even nearly 300 g $m^{-2}$), still suffer from the low *QF* level owing to high air resistance and the potential safety hazard due to electret degradation. Therefore, our novel nano-net filters stand out from all other existing HEPA filters by virtue of their superlight weight of ~80 mg $m^{-2}$ and extremely high *QF* values as a HEPA or ULPA filters, fully supporting intriguing potential applications in the fields of high-performance respirators, filter

canisters, engine intakes, appliances, medical equipment, and cleanrooms.

**Removal of PM and bacterium using 2D nano-nets.**

Supplementary Fig. 16a shows the PVDF nano-net membrane after filtrating NaCl PM. Almost all PM particles were captured and gradually accumulated on the membrane surface; even the smaller ones ($PM_{0.3}$) were effectively trapped by the networks (the inset of Supplementary Fig. 16a). Obviously, the completely covered nanofibrous networks with small pore size successfully eliminated the leaking of almost all PM particles, while having a lighter weight and thinner thickness than most of existing cutting-edge nanofibre filters. Moreover, the SEM observation of bacterium removal pre and post killing process using $TiO_2$ nano-net membrane is also conducted, the resulted images are illustrated in Supplementary Fig. 16b and c. Abundant *S. aureus* particles with diameters of ~0.8 μm were captured on the top surface of $TiO_2$ nano-net membranes. And then the bacteria were effectively killed after contacting with anatase $TiO_2$ (Supplementary Fig. 20), meanwhile, the killed-bacteria were continuously attached and filtered by the nano-nets with small pores, avoiding the re-pollution of the pathogens. More interestingly, all the membranes well maintain their structural integrity during the filtration process with high airflow or liquid stream, further confirming the robust mechanical property of the self-assembled nano-nets.

**Comparison of electrical conductivity of electrospun PAN based carbon nanofibres and carbon nano-nets.**

As illustrated in Supplementary Fig. 19, compared with most of existing electrospun PAN based carbon nanofibre membranes (1-150 S $cm^{-1}$), the single carbon nanofibre showed the increased conductivity of 100-450 S $cm^{-1}$ due to the lack of conductive path between isolated nanofibres[14-18]. In contrast, our resultant carbon nano-nets fabricated by carbonization of PAN nano-nets could achieve

electrical conductivities ranging from 180 to 750 S $cm^{-1}$, indicating the promising potential application in supercapacitors, batteries, sensors, electromagnetic interference shielding, electrostatic discharge protection.

**Crystallinity of the $TiO_2$ nano-nets.**

We have analyzed the crystalline phases of the $TiO_2$ nano-net membrane using XRD, the result is illustrated in Supplementary Fig. 20. Obviously, all the diffraction peaks could be assigned to the anatase phase of $TiO_2$, which typically has robust antibacterial capacity. Combined with the large surface area and ultrathin thickness, this anatase phase allowed the $TiO_2$ nano-nets to rapidly kill the *S. aureus* with a high killing efficiency of 99.99% while having only 1/10 weight of most existing antibacterial nanofibres[19].

## Supplementary Methods

### Simulation of electric field of the direct electronetting.

The finite element simulation of the electric field during direct electronetting was performed using COMSOL® Multiphysics software. To create the finite element analysis (FEA) model, we used the metal needle, collector, air medium and shielding net to construct the system. Simulation is based on two equations: $\nabla \cdot D=\rho_v$ and $E=-\nabla V$ ($\nabla$: Laplace operator means divergence; $D$: electric displacement field; $V$: the voltage applied; $\rho_V$: charge density on mesh; $E$: electric field), in which $D=\varepsilon_0\varepsilon_r E$ and $\varepsilon_0$ and $\varepsilon_r$ are vacuum permittivity and relative permittivity of the polymer, respectively. Using finite element axisymmetric meshing to FEA of electrostatic field, here we proposed some assumptions: 1) the dielectric constant of medium is constant and doesn't depend on electric field; 2) influences of electric charge of droplets is ignored; 3) effects of control elements on electric-field distribution would be neglected. The detailed information is listed in Supplementary Table 1.

### Optical transmittance measurement of the nano-net air filters.

The optical transmittances of the free-standing PVDF nano-net air filters were measured using an Ideaoptics IS-30-6-R integrating sphere attached to the PG 2000+ fibre optic spectrometer. The wrinkle-free nano-net filter samples were first peeled off from the PAN fibre membranes and then placed tightly on a glass slide. Afterward, we placed them in front of the integrating sphere, and the same glass slide was used as the control. Therefore, both the specular transmittance and the diffuse transmittance were included for this measurement. The transmittance spectrum was then tested by the solar spectrum from 400 to 780 nm to obtain the average transmittance.

### Filtration measurement of the nano-net air filters.

We used the LZC-K filter tester (Huada Filter Technology Co., Ltd.) to evaluate the filtration performance of the nano-net air filters. A 2 wt% NaCl aqueous solution was used to process the aerosol particles with diameters of 0.3−10 μm and a geometric standard deviation of <1.86 using the QRJ-1 NaCl atomizer. The membranes were clamped onto the filter holder with an area of 100 $cm^2$. Then, 300,000−500,000 charge-neutralized solid NaCl aerosol particles, which can be generated by the atomizer, were delivered through the testing filter by the air pump. Note that these particles were neutralized using an electrostatic neutralization device and could pass through the filter steadily and uniformly. The removal efficiencies of the filters were measured by two laser particle counters, which can detect the number of PM particles in the upstream and downstream of the particle airflow. The detection limit of this filter tester can achieve 0.0001%, meaning that filtration efficiencies of 99.9999% can theoretically be tested. To ensure the accuracy of the results, here, we recorded only data with a precision of 0.001%. The pressure drops of air filters could also be collected using two electronic pressure transducers. All tests were conducted at room temperature, 25 ± 2 °C, and a relative humidity of 40−50%.

The long-term PM purification test concerning smoke $PM_{2.5}$ and $PM_{10}$ was performed in a 0.1 $m^3$ enclosed cabin using the nano-net filter with a >99% removal efficiency and a 20 Pa pressure drop for $PM_{0.3}$ capture. Here, the model PM particles were generated by burning cigarettes and showed a broad diameter distribution from <0.3 μm to >10 μm, and most were in the range of <1 μm. Then, an artificial environment in which the air was severely polluted ($PM_{2.5}$ concentration >500 μg $m^{-3}$ and $PM_{10}$ concentration >700 μg $m^{-3}$) could be created, and the air could be fed through our nano-net filters with an area of ~25 $cm^2$ under a continuous airflow of 14 L $min^{-1}$ using an axial fan. The real value of $PM_{2.5}$ and $PM_{10}$ concentrations can be tested using a detection instrument (SDL 301, Nova Fitness). We

recorded the $PM_{2.5}$ and $PM_{10}$ concentrations once per minute until they decreased to 35 μg $m^{-3}$. The long-term recycling performance was evaluated by testing for 15 cycles.

**Cycling separation measurement of the nano-net membranes.**

To exam the reusability of our PAN nano-net membranes, a cycling separation for $TiO_2$ suspension was performed using the dead-end filtration device. With this device, the prewetted PAN nano-net membrane was fixed, and the 100 ppm $TiO_2$ nanoparticle (diameter 200–400 nm) suspension was kept pouring into the top tube to test the performance of the membranes (Supplementary Fig. 18). We recorded the initial and final rejection efficiencies and permeation fluxes of each cycle (10 min) and then backwashed the membrane with clean water 2 times before the next cycling test. The whole cycling process lasted for 50 min.

**Bioprotective activity evaluation of the nano-net membranes.**

A clinical isolate of *Staphylococcus aureus* (*S. aureus*, SA 1004) obtained from Ruby Memorial Hospital (Morgantown, WV, US) was first cultured in tryptic soy broth (TSB) for 16 h at 37 °C and then diluted with fresh TSB and cultured in at 37 °C for an additional 3 h to achieve log-phase growth. Next, 5 ml inoculum with a concentration of ~$1.0 \times 10^8$ CFU $ml^{-1}$ was fed through our $TiO_2$ nano-net membranes or electrospun $TiO_2$ nanofibre membranes, and the feed and filtrate inoculum were diluted in sterile Dulbecco's phosphate buffered saline (PBS, pH = 7.0), plated on 5% sheep blood agar plates, and incubated for 24 h at 37 °C. Then, the *S. aureus* removal efficiency could be calculated by dividing the difference between the number of colony forming units (CFUs) in the feed and that in the filtrate by the number of CFUs in the feed and then multiplying the result by 100. After air-drying for 2 min, the membrane filters used for filtering the *S. aureus* inoculum were treated in three ways: (i) immediately immersed in 3 ml PBS and sonicated for 2 min, (ii) placed under UV light of 312 nm (6

W) for 30 seconds, then immersed in 3 ml PBS and sonicated for 2 min, and (iii) placed in air in the absence of light for 30 min, then immersed in 3 ml PBS and sonicated for 2 min. Then, 400 μl medium from each of these three solutions was taken out, plated on 5% sheep blood agar plates and incubated to inspect the *S. aureus* contact killing efficiency.

## Supplementary References


1 Zhang, S., Chen, K., Yu, J. & Ding, B. Model derivation and validation for 2D polymeric nanonets: Origin, evolution, and regulation. *Polymer* **74**, 182-192 (2015).

2 Sahay, R., Teo, C. & Chew, Y. New correlation formulae for the straight section of the electrospun jet from a polymer drop. *J. Fluid Mech.* **735**, 150-175 (2013).

3 Doshi, J. & Reneker, D. H. Electrospinning process and applications of electrospun fibers. *J. Electrost.* **35**, 151-160 (1995).

4 Gamero-Castaño, M. & Magnani, M. Numerical simulation of electrospraying in the cone-jet mode. *J. Fluid Mech.* **859**, 247-267 (2019).

5 Rosell-Llompart, J., Grifoll, J. & Loscertales, I. G. Electrosprays in the cone-jet mode: From Taylor cone formation to spray development. *J. Aerosol Sci.* **125**, 2-31 (2018).

6 Gamero-Castano, M. Energy dissipation in electrosprays and the geometric scaling of the transition region of cone–jets. *J. Fluid Mech.* **662**, 493-513 (2010).

7 Persano, L. *et al.* High performance piezoelectric devices based on aligned arrays of nanofibers of poly (vinylidenefluoride-co-trifluoroethylene). *Nat. Commun.* **4**, 1633 (2013).

8 Wang, X., Ding, B., Yu, J. & Wang, M. Engineering biomimetic superhydrophobic surfaces of electrospun nanomaterials. *Nano Today* **6**, 510-530 (2011).

9 Hou, L. *et al.* Bioinspired superwettability electrospun micro/nanofibers and their applications. *Adv. Funct. Mater.* 1801114 (2018).

10 Zhang, S. *et al.* A controlled design of ripple-like polyamide-6 nanofiber/nets membrane for high-efficiency air filter. *Small* **13**, 1603151 (2017).

11 Zuo, F. *et al.* Free-standing polyurethane nanofiber/nets air filters for effective PM capture. *Small* **13**, 1702139 (2017).

12 Zhang, S. *et al.* Tailoring mechanically robust poly(m-phenylene isophthalamide) nanofiber/nets for ultrathin high-efficiency air filter. *Sci. Rep.* **7**, 40550 (2017).

13 Li, P., Wang, C., Zhang, Y. & Wei, F. Air filtration in the free molecular flow regime: A review of high-efficiency particulate air filters based on carbon nanotubes. *Small* **10**, 4543-4561 (2014).

14 Zhang, B., Kang, F., Tarascon, J. M. & Kim, J. K. Recent advances in electrospun carbon nanofibers and their application in electrochemical energy storage. *Prog. Mater. Sci.* **76**, 319-380 (2016).

15 Sharma, C. S., Katepalli, H., Sharma, A. & Madou, M. Fabrication and electrical conductivity of suspended carbon nanofiber arrays. *Carbon* **49**, 1727-1732 (2011).

16 Inagaki, M., Yang, Y. & Kang, F. Carbon nanofibers prepared via electrospinning. *Adv. Mater.* **24**, 2547-2566 (2012).

17 Ge, J. *et al.* Elastic and hierarchical porous carbon nanofibrous membranes incorporated with $NiFe_2O_4$ nanocrystals for highly efficient capacitive energy storage. *Nanoscale* **8**, 2195-2204 (2016).

18 Im, J. S., Kang, S. C., Lee, S. H. & Lee, Y. S. Improved gas sensing of electrospun carbon fibers based on pore structure, conductivity and surface modification. *Carbon* **48**, 2573-2581 (2010).

19 Si, Y. *et al.* Daylight-driven rechargeable antibacterial and antiviral nanofibrous membranes for bioprotective applications. *Sci. Adv.* **4**, eaar5931 (2018).
